# Lactoferrin docking NIR-II cyanine dye as a potentiated phototheranostic for synchronous multimodal bioimaging and tumor photo-immunotherapy

**DOI:** 10.7150/thno.102663

**Published:** 2024-10-14

**Authors:** Lifeng Hang, Haijian Li, Meng Li, Yiqiang Sun, Wenjiao Wu, Laiping Fang, Yanzhao Diao, Hong Qu, Tao Zhang, Shumei Li, Guihua Jiang

**Affiliations:** 1 The Department of Medical Imaging, Guangzhou Key Laboratory of Molecular Functional Imaging and Artificial Intelligence for Major Brain Diseases, The Affiliated Guangdong Second Provincial General Hospital of Jinan University, Guangzhou 518037, P. R. China.; 2 School of Chemistry and Chemical Engineering, University of Jinan, Jinan 250022, P. R. China.; 3 School of Medicine, Jinan University, Guangzhou, 510632, P. R. China.; 4 School of Physical and Mathematical sciences, Nanyang Technological University, 21 Nanyang Link, Singapore, 637371, Singapore.

**Keywords:** Lactoferrin, Bovine serum albumin, Multimodal imaging, NIR-II phototherapy, Immunogenic cell death, Photo-immunotherapy

## Abstract

**Rationale:** A promising dye for phototheranostics, IR-1048 is a near-infrared region II (NIR-II) cyanine dye that exhibits exceptional optical characteristics in NIR-II spectrum. Unfortunately, the biological applications of IR-1048 are challenged by its hydrophobic nature, the formation of face-to-face stacked dimeric aggregates (H-aggregates) that result in pronounced spectral blue shifts, and issues related to fluorescence quenching.

**Method:** We present a novel docking strategy involving bovine serum albumin (BSA) and lactoferrin (Lf) to construct BSA@IR-1048 and Lf@IR-1048 nanoprobes. The NIR-II optical characteristics of these nanoprobes have been thoroughly investigated through both theoretical and experimental approaches. In addition, we conducted *in vitro* and* in vivo* evaluations of their NIR-II photothermal and photodynamic properties, multimodal imaging capabilities, and effectiveness in photoimmunotherapy.

**Results:** Following the protein docking process, both BSA@IR-1048 and Lf@IR-1048 probes exhibited a red-shifted absorbance peak and an "ON" state in NIR-II fluorescence. Theoretical analyses alongside experimental results indicate that Lf@IR-1048, which has a higher docking binding energy of -10.83 kcal/mol, significantly enhances optical characteristics in the NIR-II region. Notably, when utilizing a single NIR-II light source, Lf@IR-1048 was effective in producing single-linear state oxygen and converting photons into heat energy, achieving a photo-thermal conversion efficiency of 41.9%. The overexpression of transferrin receptors in tumor cells also improved tumor-targeting and enrichment capabilities of Lf@IR-1048, as demonstrated* vitro* and* in vivo* studies. Comparatively, Lf@IR-1048 facilitated multimodal imaging-guided NIR-II phototherapy, showing an impressive tumor development inhibition rate of 94.8%. Furthermore, in bilateral CT26 tumor-bearing mice, the Lf@IR-1048-based photo-immunotherapy exhibited significant antitumor activity, attributed to enhanced dendritic cell maturation and infiltration of cytotoxic T lymphocytes.

**Conclusion:** Lf@IR-1048 displays a powerful combination of photothermal therapy, photodynamic therapy, and tumor-targeting potential for effective multimodal imaging-guided NIR-II phototherapy, leading to substantial inhibition of tumor growth.

## Introduction

Significant attention has been directed towards near-infrared region II (NIR-II) cancer photo-immunotherapy, encompassing both photodynamic immunotherapy and photothermal immunotherapy. These therapeutic modalities are characterized by low drug resistance, minimal tissue absorption, deeper tissue penetration, real-time diagnostic capabilities, and least invasiveness 1-4. Photothermal therapy (PTT) employs a photothermal agents to destroy cancer cells upon exposure NIR-II light, while photodynamic therapy (PDT) utilizes photosensitizers (PS) to trigger apoptosis and generate lethal reactive oxygen species (ROS) 5-8. Furthermore, damage-associated molecular patterns (DAMPs) such as calreticulin (CRT), adenosine triphosphate (ATP), and high mobility group box 1 (HMGB1) are released during immunogenic cell death (ICD) initiated by PDT and PTT, which can elicit immunological responses 9-12. Additionally, the PS can guide treatment progression through photoacoustic imaging and fluorescence monitoring 13. However, the necessity of using distinct PDT and PTT molecules complicates the therapeutic approach. To further enhance photo-immunotherapy, the development of NIR-II PS with strong ROS generation and photothermal capabilities is essential.

Cyanine dyes, due to their highly tunable structures, readily absorb and emit wavelengths within the near-infrared spectrum 14-17. A notable NIR-II PS, IR-1048, demonstrates favorable optical characteristics, absorbing light in the range of 900 nm to 1060 nm, indicating its potential for phototheranostics 18. Nonetheless, the hydrophobic nature, inadequate photostability, unpredictable pharmacokinetics, and limited water solubility of IR-1048 restrict its biological application. Previous studies have shown that planar cyanine dye exhibits significant π-π stacking in aqueous solution, leading to the formation of face-to-face stacked dimeric aggregates (H-aggregates) that display marked fluorescence quenching and spectral blue shifts compared to monomer absorption 19. To enhance both water solubility and photostability, researchers incorporated hydrophilic 2-nitroimidazole into IR-1048, which significantly improved the water solubility of probe. However, the couping of 2-nitroimidazole proved challenging and resulted in only marginal enhancements in water solubility 20. Geng *et al.* proposed utilizing liposomes to load IR-1048 effectively for targeted delivery to tumor sites during phototherapy 21. However, its efficacy in photo-immunotherapy and NIR-II phototheranostics is constrained by the undocumented PDT property and reduced fluorescence intensity.

To enhance *in vivo* biological activity, exogenous and endogenous albumin-encapsulated small-molecule cyanine dyes have been explored in prior studies 22-24. Sulfhydryl-containing proteins can form covalent bonds with cyanine dyes containing chlorine, effectively stabilizing the dyes within the hydrophobic pocket of the proteins 25-28. This encapsulation significantly increases the physiological brightness of the dye molecules by reducing non-radiative transitions caused by internal rotations and vibrations 29. For example, the preferred conformation of IR-780 within the albumin pocket promotes twisted intramolecular charge transfer, resulting in enhanced NIR-II emission 30. Liu's team developed nanocomposites by combining human serum albumin (HSA) with various NIR dyes, leading to enhanced chemical stability through cross-linking 31. Additionally, Yuan's group encapsulated heavy atom and alkyl chain-modified BODIPY derivatives within reduced bovine serum albumin (BSA), demonstrating good biocompatibility, stability, and a photothermal conversion efficiency of up to 58.7% 32. Inspired by these findings, we aimed to improve the water solubility and photostability of IR-1048 while screening for new proteins with enhanced NIR-II optical characteristics. Consequently, we developed NIR-II phototheranostic protein@IR-1048 systems for PDT and PTT-mediated photo-immunotherapy.

**Scheme 1:**
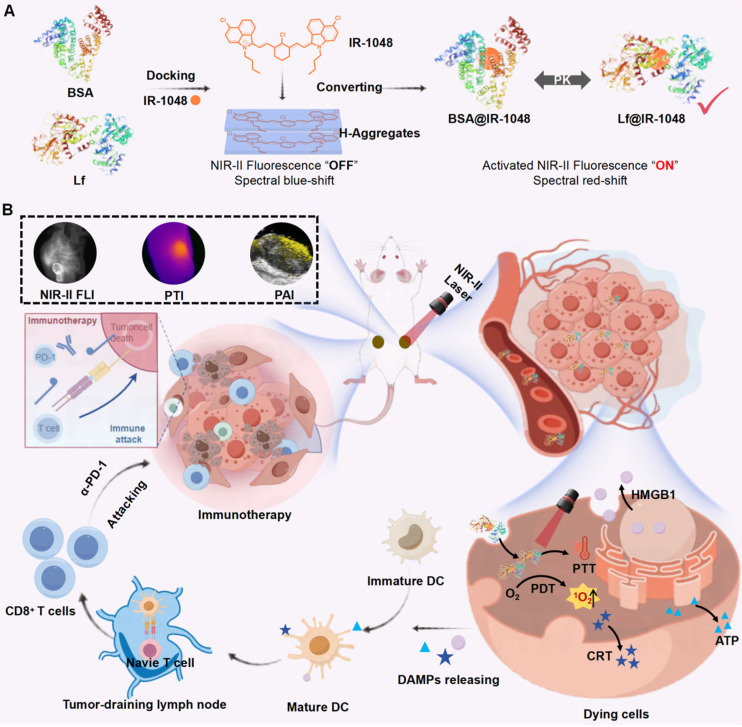
**(A)** The schematic diagram showing the synthesis process of Lf with BSA proteins hitchhiking IR-1048. **(B)** The schematic diagram showing Lf@IR-1048 for multimodal bioimaging and NIR-II phototherapy induced ICD combined with PD-1 antibody to achieve tumor photoimmunotherapy.

In our study, we selected bovine serum albumin (BSA, 66.4 kDa) and lactoferrin (Lf, 80 kDa) due to their distinct biological properties. Cyanine dyes can be stably incorporated into the hydrophobic pocket of BSA, which is known for its long blood half-life, stability and biocompatibility 33, 34. Lf, a naturally occurring glycoprotein involved in iron transport, can internalize into tumor cells to various overexpressed receptors, such as low-density lipoprotein and transferrin receptors 35-37. We devised a strategy involving BSA and Lf to construct BSA@IR-1048 and Lf@IR-1048 nanoprobes, as illustrated in **Scheme 1A**. The IR-1048 dye exhibited a blue-shifted absorbance spectrum peak, attributed to its H-aggregate formation in its fluorescence "OFF" state. As anticipated, upon the docking of proteins to the molecule, the BSA@IR-1048 and Lf@IR-1048 probes displayed a red-shifted absorbance peak and activated NIR-II fluorescence. Moreover, protein encapsulation enhanced the overall biosafety and hydrophilia of both nanoprobes. Notably, Lf@IR-1048, with a higher docking binding energy of -10.83 kcal/mol, significantly improved optical characteristics in the NIR-II region compared to BSA@IR-1048. Lf@IR-1048 exhibited superior NIR-II fluorescence brightness and photothermal conversion efficiency (PTCE) relative to its BSA@IR-1048. The Lf hitchhiking mechanism enables the Lf@IR-1048 to function more efficiently and easily, generating single-linear state oxygen (^1^O_2_) and converting photons into thermal energy using a single 1064 nm laser. Additionally, both *in vitro* and *in vivo* studies demonstrated that Lf@IR-1048 exhibited enhanced tumor targeting and accumulation (**Scheme 1B**). Compared to the control group, Lf@IR-1048 mediated NIR-II phototherapy effectively inhibited tumor growth by 94.8%. Moreover, in bilateral CT26 tumor-bearing mice, Lf@IR-1048-based NIR-II photo-immunotherapy exhibited more pronounced antitumor effect, correlating with the release of DAMPs from dying cancer cells. Tumor-draining lymph node cells utilize tumor-associated antigens from the primary tumor to stimulate dendritic cell (DC) maturation and mount a robust immune response. Following photo-immunotherapy, cytotoxic T cells were found to infiltrate the bilateral tumors effectively. The red-shifted absorbance peak and activated NIR-II fluorescence of Lf@IR-1048 can be leveraged for photoacoustic imaging-guided tumor photo-immunotherapy. Collectively, these studies present a viable approach to docking NIR-II cyanine dyes onto other proteins to facilitate multimodal imaging-guided tumor NIR-II photo-immunotherapy.

## Methods

### Reagents

We obtained the following from Sigma-Aldrich: 2',7'-Dichlorodihydrofluorescein diacetate (DCFH-DA), anhydrous dimethyl sulfoxide (DMSO), triacetonamine (TEMP), 1,3-Diphenylisobenzofuran (DPBF), Cy3, methylene blue (MB), dihydrorhodamine 123 (DHR123) N-Acetylcysteine amide (NACA), IR-1048, Lactoferrin (Lf, ≥ 98%), and bovine serum albumin (BSA, ≥ 98%). Gibco BRL (Eggenstein, Germany) supplied Roswell Park Memorial Institute (RPMI) 1640, Dulbecco's modified eagle medium (DMEM), and fetal bovine serum (FBS). Anti-PD-1 was purchased from Selleck. The Milli-Q System (Millipore, Bedford, MA, USA) provided the Milli-Q water (18.2 MΩ·cm).

### Characterization

The ^1^H nuclear magnetic resonance (NMR) and ^13^C NMR spectra were obtained on a Bruker AM-400 spectrometer. A background-corrected TP-720 spectrophotometer was used to measure the optical absorption spectra of various probes. The FS5C fluorescence spectrophotometer was used to measure the NIR-II fluorescence spectra of several probes. A BIO-RAD electrophoresis machine was used to carry out sodium dodecyl sulfate-polyacrylamide gel electrophoresis (SDS-PAGE). Using a Bruker Ultraflextreme MALDI-TOF, the bonding behavior of Lf/BSA with IR-1048 was studied. Room temperature dynamic light scattering (DLS) examination was carried out with a Malvern Zetasizer nano ZS size analyzer. Using an FEI Talos F200x, transmission electron microscopy (TEM) pictures were captured. All samples' Fourier Transform Infrared (FTIR) spectra were measured using a Nicolet iS 10. Bruker EMXplus electron spin resonance (ESR) spectrum of ^1^O_2_.

### Synthesis of Lf@IR-1048, BSA@IR-1048, and Lf@Cy3

Initially, DMSO and aqueous solution containing 4 mM IR-1048 and 25 µM Lf, respectively, were produced. Then, 40 mL of a 25 µM Lf solution was mixed vigorously with 250 µL of 4 mM IR-1048 using a fast vortex (the molar ratio of protein and dyes was 1:1). The Lf@IR-1048 was then produced by reacting the combination solution for two hours at 50 °C. In order to use Lf@IR-1048* in vivo*, it was finally ultrafiltered to a concentration of 500 µM.

In DMSO and aqueous solution, 4 mM IR-1048 and 25 µM BSA were created, respectively. Then, 250 µL IR-1048 (4 mM) was added into 40 mL BSA solution (25 µM), and the mixture was sufficiently mixed by quickly vortexing (the molar ratio of protein and dyes was 1:1). The BSA@IR-1048 was then produced by reacting the combination solution for two hours at 50 °C. Ultimately, the BSA@IR-1048 was ultrafiltered concentrated to 500 µM.

50 µL Cy3 (10 mM) was added into 20 mL Lf solution (25 µM), and the mixture was sufficiently mixed by quickly vortexing (the molar ratio of protein and dyes was 1:1). The Lf@Cy3 was then produced by reacting the combination solution for two hours at 50 °C. Ultimately, the Lf@Cy3 was ultrafiltered concentrated to 1 mM.

### Photothermal property of IR-1048, Lf@IR-1048, and BSA@IR-1048

Prior to being continuously exposed to a 1064 nm laser (0.5 W/cm^2^) for five minutes, the aqueous solutions of IR-1048, Lf@IR-1048, and BSA@IR-1048 (20 μM) were placed into centrifuge tubes, respectively. The Thermal Imaging Camera (FOTRIC 600C) was used to track the temperature. Subsequently, solutions with varying Lf@IR-1048 concentrations (0, 5, 10, and 20 μM) were exposed to a 1064 nm laser at a power density of 0.5 W/cm^2^ for a duration of 5 minutes. Assays using laser on/off cycles were used to assess the photothermal stability of Lf@IR-1048. A 1064 nm laser (0.5 W/cm^2^) was applied to the IR-1048, BSA@IR-1048, and Lf@IR-1048 aqueous solution (10 μM) for 2.5 minutes, and then the laser was turned off for a further 2.5 minutes. Six cycles of a four-minute interval were employed to turn the laser on and off. Furthermore, the values of the photothermal conversion efficiency (PTCE) were computed using the formula from the earlier report 38.

### The detection of singlet oxygen

To ascertain the PDT responses of each sample (IR-1048, Lf@IR-1048, and BSA@IR-1048), DCFH-DA was used as the ROS probe. The solutions IR-1048, Lf@IR-1048, and BSA@IR-1048, which included 10 μM IR-1048 and 10 μM DCFH-DA, were exposed to a 1064 nm laser for five minutes at a power of 0.5 W/cm^2^. The ROS production was then determined by measuring the fluorescence intensity at 504 nm excitation and 529 nm emission, and the fluorescence emission spectra of DCF were displayed at one-minute intervals.

The application of ESR measurement allowed for the detection of ^1^O_2_ production via TEMP. Once exposed to a 1064 nm laser (0.5 W/cm^2^) for five minutes, TEMP and nanoparticles were mixed with D_2_O aqueous to detect the ESR spectra of ^1^O_2_ in each group. Additionally, the production of ^1^O_2_ was investigated by tracking the spectral shift of the DPBF probe when exposed to a 1064 nm laser (0.5 W/cm^2^). Next, the absorption spectra of DPBF were measured. To further analyze the type of reactive oxygen species, MB and DHR123 were used to detect hydroxyl radicals and superoxide radicals, respectively. The absorption spectra of MB probes were recorded after laser irradiation with or without Lf@IR-1048. Next, the fluorescence spectra of DHR123 (λex: 495 nm and λem: 525 nm) were measured after laser irradiation with or without Lf@IR-1048.

### Cell culture

The colon cancer cell line CT26 was cultivated in RPMI-1640, for the research. 10% FBS, 100 units/mL penicillin, and 100 µg/mL streptomycin were added to each medium. The cells were cultivated in an atmosphere containing 5% CO_2_ at 37 °C. The normal cell line Beas-2B cells was cultured in DMEM culture medium supplemented with 10% FBS and antibiotics at 37 °C under a humidified atmosphere containing 5% CO_2_.

### Cell uptake studies

Confocal dishes were injected with 1 × 10^5^ CT26 cells at 37 °C. A fresh medium containing BSA@IR-1048 and Lf@IR-1048 (20 μM IR-1048) was added after 24 hours. Following an incubation period of 1, 2, 4, 6, and 8 hours, the cells were fixed in 4% paraformaldehyde, and pictures were captured using an NIR-II microscope (MARS-micro full spectrum). CT26 cells were pretreated with or without Anti-TfR1 (100 μM) for 4 h, then incubated with Lf@Cy3 complex for 6 h. The inhibition of cell uptake of Lf@Cy3 complex were then analyzed by fluorescent microscope. Beas-2B cells were incubated with Lf@Cy3 complex for 6 h, and the cell uptake of Lf@Cy3 complex were then analyzed by fluorescent microscope.

### Cell viability assays

The conventional CCK-8 test was used to assess cell viability. After inoculating CT26 and Beas-2B cells in 96-well plates (5 × 10^3^ cells per well), the cells were treated with varying doses of Lf@IR-1048 for a duration of 48 hours. Next, 10 μL of CCK-8 was added to each well in a 96-well plate. After incubating for 2 h, the absorbance of cells at 450 nm was recorded using a microplate reader (Thermo Fisher).

After inoculating CT26 and Beas-2B cells in 96-well plates (5 × 10^3^ cells per well), the cells were treated for 6 hours with IR-1048 (20 μM) and Lf@IR-1048 (20 μM). The cells were incubated at 37 °C for an additional 24 hours after being subjected to the NIR-II laser for 5 minutes (1064 nm, 0.5 W/cm^2^) at either room temperature or adding ROS quenching agent (NACA: 1 mM). Then, 10 μL of CCK-8 was added to each well in a 96-well plate for 2 h. Ultimately, a microplate reader was used to record the absorbance of cells at 450 nm to calculate cell viability.

CT26 cells were inoculated in 24-well plates (1 × 10^5^ cells per well) and then treated with PBS, only laser, Lf@IR-1048, IR-1048 + L, Lf@IR-1048 + L (20 μM), and Lf@IR-1048 + L + NACA for the purpose of live/dead cell double labeling. The cells were exposed to an NIR-II laser (1064 nm, 0.5 W/cm^2^, 5 min) and further cultivated for six hours. All groups were then stained with calcein-AM/7-AAD solution. Using a flow cytometer (BD FACS Aria III) and an Olympus BX51 fluorescent microscope, live and dead cells were examined.

### Intracellular ROS production efficiency

CT26 cells were inoculated in 24-well plates (1 × 10^5^ cells per well), incubated for 24 hours, and then new media containing 20 μM IR-1048, Lf, and Lf@IR-1048 solution was added to analyze ROS generation in cells. After six hours of incubation, the cells were exposed to an NIR-II laser for five minutes (1064 nm, 0.5 W/cm^2^). Subsequently, the treated cells were assessed using a fluorescence microscope after being stained with DCFH-DA (1 μM).

### The detection of ATP, CRT, and HMGB1

In 24-well plates, 1 × 10^5^ cells were seeded per well to cultivate CT26 cells for a whole day. After being inserted, the Lf@IR-1048 was exposed to 5 minutes of 1064 nm laser radiation (0.5 W/cm^2^). After an additional 8 hours of incubation, the absorbance at 450 nm was measured using a microplate reader, and extracellular ATP, CRT, and HMGB1 in the conditioned medium produced from treated cells were evaluated using Enzyme-Linked Immunosorbent Assay (ELISA).

Subsequently, the treated cells underwent a 30-minute incubation period with distinct primary antibodies against HMGB1 and CRT. The exposure of HMGB1 and CRT was then determined using Leica TCS SP8 laser scanning confocal microscopy.

### *In vitro* determination of DC maturation

Briefly, CT26 cells and BMDCs were separately seeded in a 24-well Transwell culture plate's upper layer or lower layer and cultured for 24 h. After co-incubated with different groups for 24 h, the DCs were collected and labeled by using the following anti-mouse antibodies: anti-CD11c-APC, anti-CD80-PE, and anti-CD86-FITC (Biolegend). After PBS washing, the cells were analyzed via flow cytometry.

### Animal and tumor model

Six-week-old female Balb/c mice were acquired from Zhuhai Biotest. All procedures were authorized by the Second Guangdong General Hospital's Animal Care and Use Committee (Approval No. 2023-DW-KZ-130-02), and the animals were maintained in accordance with the parameters outlined in the Guide for the Care and Use of Laboratory Animals. One hundred microliters of 1 × 10^6^ CT26 cells were subcutaneously injected to create the xenograft model.

### *In vivo* imaging and pharmacokinetic studies

The NIR-II *in vivo* imaging system (MARS) was used to capture the *in vivo* NIR-II fluorescence images. For *in vivo* NIR-II fluorescence imaging, intratumoral injections of BSA@IR-1048 or Lf@IR-1048 (IR-1048: 2 mg/kg) were made into CT26 tumors once the tumor volumes had grown to ~200 mm^3^. Before imaging, all mice were shaved with depilatory lotion and given isoflurane anesthesia. The mice were injected, then they were placed on the imaging table and exposed to an 880 nm laser at 6, 12, 24, and 48 hours later. Major organs such as the heart, liver, spleen, lung, kidney, and tumor tissue were removed 48 hours after injection, and the NIR-II fluorescence imaging system was also used to record the images. ImageJ software was used to analyze every fluorescence image. 20 µL blood samples were taken from the mouse retro-orbital plexus at pre-arranged intervals (0.5, 1, 2, 4, 8, 12, 24, and 36 hours) for the pharmacokinetics investigation. The NIR-II fluorescence imaging equipment was used to measure the fluorescence intensity at various time points.

The Lf@IR-1048 dispersions were then scanned using an ultra-high resolution small animal photoacoustic multimode imaging system (Vevo LAZR-X) at different concentrations (0, 1.25, 2.5, 5, 10, and 20 μM). Lf@IR-1048 dispersion was administered intravenously into CT26 tumor-bearing mice for *in vivo* photoacoustic (PA) imaging. A scan was conducted 12 hours following injection.

### Photothermal effects of Lf@IR-1048* in vivo*

Mice carrying tumors of CT26 cells were given injections of Lf@IR-1048 (IR-1048: 2 mg/kg) and exposed to laser radiation (1064 nm, 0.5 W/cm^2^) for five minutes at the tumor location. PBS-injected mice served as the negative control group. Using an infrared thermal camera, the temperature and pictures at the tumor site were captured concurrently.

### Anti-tumor activity of Lf@IR-1048 *in vivo*

Five groups (n = 5) consisting of female balb/c mice aged six to eight weeks were randomly assigned: (1) PBS group; (2) PBS + laser group; (3) Lf@IR-1048 group; (4) IR-1048 + laser group; and (5) Lf@IR-1048 + laser group. At the start of the experiment, each animal received a subcutaneous injection of 1 × 10^6^ CT26 cells, and the tumors were allowed to grow to a size of 80 mm^3^. Intravenous injections of Lf@IR-1048, IR-1048 (at a dose of IR-1048: 2 mg/kg), or an equivalent volume of saline were given to each group. Twelve hours after injection, mice in the PBS, Lf@IR-1048, and IR-1048 groups were exposed to a laser (1064 nm, 0.5 W/cm^2^, 5 min). Every three days, the body weight and tumor volume were measured. The formula used to compute the tumor volume was V= L×W^2^/2 (mm^3^), where L stands for the longest dimension and W for the shortest. Tumors were removed at the conclusion of treatment and stained with Hematoxylin and Eosin (H&E), Ki67, and TdT-mediated dUTP Nick-End Labeling (TUNEL).

Blood samples, including those for erythrocytes, leukocytes, platelets, hemoglobin, aspartate transaminase (AST), alanine transaminase (ALT), and creatinine (CREA), were drawn for standard blood tests and biochemical analyses. Following mouse dissection, the main organs were preserved in 4% formalin. Following a 24-hour fixation period, tissues were sectioned, stained with H&E for histological assessment, and typically embedded in important organs such as the heart, liver, spleen, lungs, and kidneys.

### Anti-tumor activity and immune response in bilateral CT26 tumor-bearing mice

To replicate the initial tumor, CT26 cells (1 × 10^6^) were subcutaneously injected into the right side of each mouse. Each mouse had a subcutaneous injection of CT26 cells to mimic a distant tumor six days later. Afterwards, these mice were split into 5 groups (n = 3) at random: (1) PBS group, (2) Lf@IR-1048 group, (3) Anti-PD-1 group, (4) Lf@IR-1048 + laser group, and (5) Lf@IR-1048 + laser + anti-PD-1 group. Intravenous injections of saline, anti-PD-1 (20 μg), and Lf@IR-1048 (IR-1048: 2 mg/kg) were administered to mice at the previously mentioned doses. Twelve hours following injection, laser stimulation was carried out. Every two days, measurements of body weight and tumor volume were made. Mice blood samples were obtained after the conclusion of treatments in order to evaluate TNF-α, IL-6, and IFN-γ. Flow cytometry was used to identify the maturation of DCs in tumor-draining lymph node cells that had been extracted and labeled with anti-CD11c-APC, anti-CD80-PE, and anti-CD86-FITC (Biolegend).

The distant tumor-draining lymph node cells were extracted and stained with anti-CD45-BV510, anti-CD3-FITC, anti-CD4-APC, anti-CD8a-APC/Cy7, anti-CD69-PE/Cy7, and anti-CD44-PerCP/Cy5.5 (Biolegend) in accordance with the manufacturer's instructions in order to more accurately assess the immune response *in vivo*. To find the percentage of cytotoxic T lymphocytes, cells were stained with fluorescence-labeled antibodies and subjected to FACS analysis. Sections of bilateral CT26 tumors were removed and treated with CD8a antibody (green fluorescence) in order to examine the immune cells within the tumors. Afterwards, CLSM saw them after they had been dyed with DAPI.

### Statistical analysis

GraphPad Prism was used for statistical analysis. The data is expressed as mean ± SD. For every statistical analysis, the sample size (n) exceeded three. One-way ANOVA and Tukey's post hoc test were used to examine group differences; *p < 0.05, **p < 0.01, ***p < 0.001, **** p < 0.0001.

## Results and Discussion

### Synthesis and characterization of protein@IR-1048 probes

**Figure 1:**
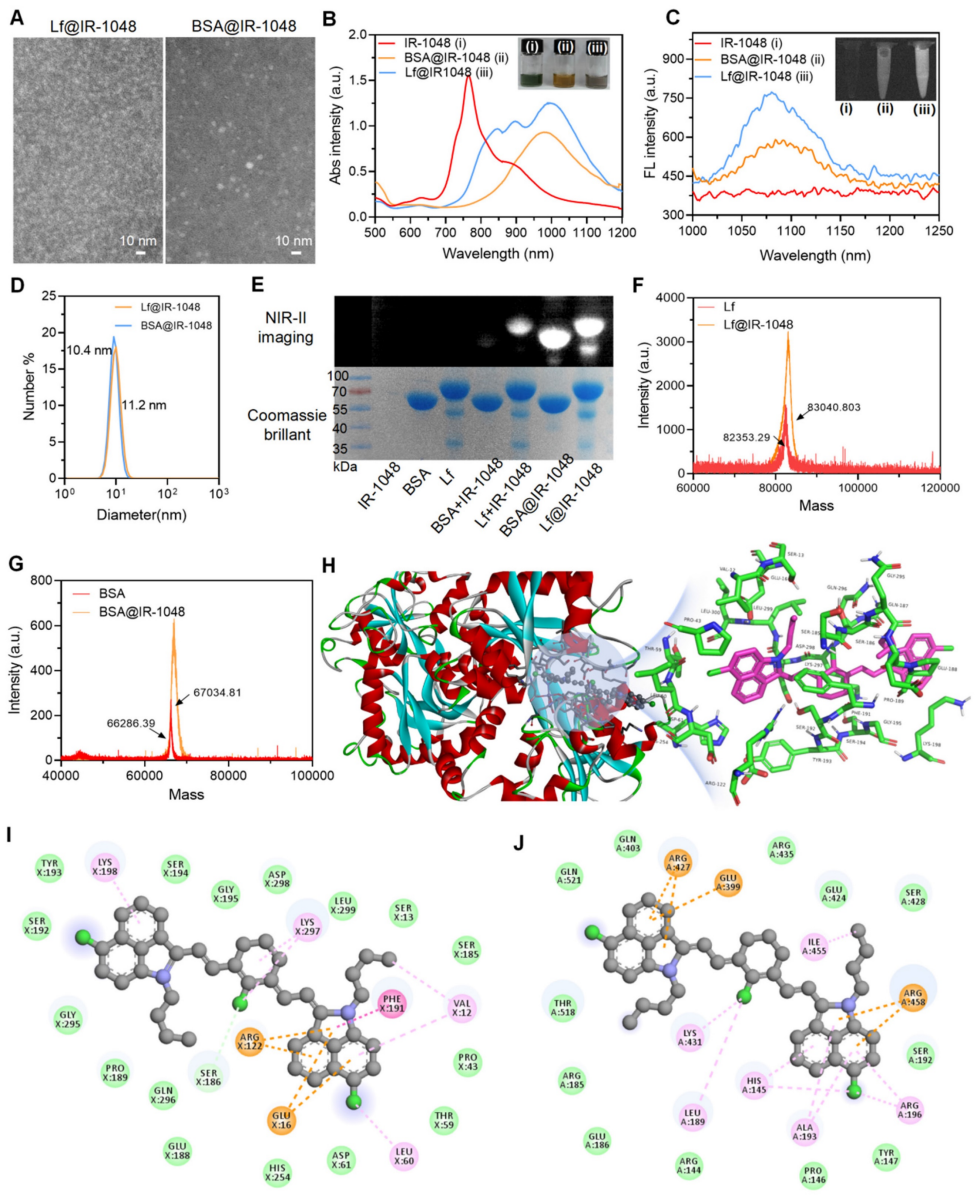
** Characterization of protein@IR-1048 probes**. **(A)** TEM images of Lf@IR-1048 and BSA@IR-1048 nanoparticles. **(B)** UV-vis-NIR spectra of IR-1048, BSA@IR-1048, and Lf@IR-1048 in water, and corresponding sample aqueous solutions in the insets. **(C)** Fluorescence spectra of IR-1048, BSA@IR-1048, and Lf@IR-1048 in water under excitation wavelength of 980 nm. **(D)** The hydrodynamic sizes of BSA@IR-1048 and Lf@IR-1048. **(E)** The SDS-PAGE and NIR-II fluorescence images of BSA and Lf covalent binding with IR-1048. **(F)** High-resolution mass spectrometry of BSA and BSA@IR-1048. **(G)** High-resolution mass spectrometry of Lf and Lf@IR-1048. **(H)** Theoretical simulations of IR-1048 binding to Lf protein in the gliding docking mode. **(I)** Interactions between IR-1048 and Lf residues. **(J)** Interactions between IR-1048 and BSA residues.

One small hydrophobic compound with good NIR-II optical characteristics that is sold commercially is IR-1048 dye. Prior research has demonstrated that planar elanine exhibits robust π-π stacking in aqueous solution, resulting in the formation of H-aggregates that exhibit the greatest spectral blue shifts and fluorescence quenching in comparison to the corresponding monomer absorption 19. Hydrophilicity and photostability are significantly increased when IR-1048 dye binds to the hydrophobic pocket of proteins, enhancing the intramolecular charge transfer mechanism. First, we carried out ^1^H NMR, ^13^C NMR, and electron impact mass spectrometry (EI-MS) to demonstrate the desired reagent of IR-1048. As shown in **Figure S1**, the ^1^H NMR, ^13^C NMR, and EI-MS data prove that the used reagent was IR-1048 20. Then, we have successfully built two stable protein@cyanine dye probes (Lf@IR-1048 and BSA@IR-1048) based on the mechanism of cyanine dye binding to proteins. The resulting probes, which are not substantially different from the original protein morphology, have a uniform morphology with homogenous sizes of 8 nm and 7 nm in Lf@IR-1048 and BSA@IR-1048, respectively, as shown in **Figure 1A** and **S2**. The fluorescent "OFF" IR-1048 has a blue-shifted absorption peak at 764 nm in PBS (pH 7.4) as a result of its H-aggregation. The absorbance peaks of IR-1048 rapidly red-shifted to around 1000 nm upon docking to Lf and BSA, concurrently with a reduction in the absorbance peak at 764 nm (**Figure 1B**). When IR-1048 H-aggregates attach to the protein, they shift from being monomers to 764 nm and 1000 nm in absorption. The inset of **Figure 1B** displays the matching homogenous solutions of distinct colors, demonstrating the successful production of two stable protein@cyanine dyes. Furthermore, there is a strong linear correlation between the concentration and the maximum absorbance of the protein@IR-1048 probes (**Figure S3** and **S4**). At a wavelength of 1064 nm, Lf@IR-1048 had a molar extinction coefficient of 6.43 × 10^4^ M^-1^cm^-1^, which is higher than that of BSA@IR-1048 (4.58 × 10^4^ M^-1^cm^-1^) and IR-1048 (8.42 × 10^2^ M^-1^cm^-1^). This suggests that Lf@IR-1048 has a greater capacity to absorb light in the NIR-II area. The fluorescence intensity of IR-1048 coupled to BSA and Lf increased by one and two times, respectively, as validated by direct imaging using an InGaAs camera excited at 980 nm (**Figure 1C**). The fluorescence intensity of Lf@IR-1048 exhibited a concentration dependence, as seen in **Figure S5**. Furthermore, in comparison to the free protein, the hydrated particle sizes of both protein@cyanine dyes rose by roughly ~1 nm (**Figure 1D** and **S6**). The protein@cyanine dyes retain their negative potentials even after binding to Lf and BSA, suggesting that they are suitable for biological applications (**Figure S7**).

The development of the Lf@IR-1048 and BSA@IR-1048 complex was further confirmed by localizing the protein@cyanine dyes using SDS-PAGE, NIR-II imaging, and Caumas Brilliant Blue staining. The fluorescent band of IR-1048 changed to around 66.5 kDa and 80 KDa after docking to BSA and Lf, overlapping with the protein bands fully, showing that IR-1048 was encapsulated in BSA and Lf, in contrast to free IR-1048 (**Figure 1E**). A protein molecule exclusively binds to one dye, according to the results of high-resolution mass spectrometry (**Figure 1F** and **1G**). Subsequently, FTIR studies were performed on all samples to further demonstrate the successful docking of molecules to proteins. Additional vibrational peaks (1097.5 cm^-1^ and 663 cm^-1^) appeared in the FTIR spectra of both proteins after docking the IR-1048 molecule compared to the FTIR spectra of BSA and Lf (**Figure S8**). The peaks at 1097.5 cm^-1^ and 663 cm^-1^ were respective assigned to the C-H out-of-plane bending vibration and δCH of benzene ring in IR-1048, suggesting that the protein@IR-1048 probes were successfully prepared.

Based on previous reports on the docking of cyanine dyes to proteins, there are three main stages 23: (I): a cyanine dye molecule is inserted into the hydrophobic pocket of the protein; (II) the cysteine-SH group and the Cl-C group of the cyanine dye molecule form a covalent bond; (III) the covalent conformation between the dye molecule and the protein is fine-tuned to achieve the final steady-state luminescent NIR fluorescence. However, the docking of Lf with IR-1048 only converts the face-to-face stacked dimers (H-aggregation) into molecular monomers, which restores the originally quenched fluorescence with no enhancement. We used non-covalent molecular docking simulations using a gliding software to gain a better understanding of the binding mechanisms of the Lf and BSA proteins to IR-1048. Software simulates the molecular docking models to depict molecular-protein interactions, as demonstrated in **Figure 1H** and **S9**. **Figure 1I** and **1J** show the two-dimensional pictures enabling a clearer view of the interactions between the IR-1048 residue and the proteins BSA and Lf. More interactions take place between IR-1048 and the amino acid residues of Lf: π-π interaction (Phe191), anion-π interaction (Glu16), cation-π interaction (Arg122), ligand are alkyl-halogen interaction (Leu60), alkyl- and alkyl-chloro interaction (Lys297), cation-π interaction (Lys198), π-π interaction (Val12), π-π interaction (Arg122), anion-π interaction (Glu16), and cation-π interaction (Lys198).

However, only four types interactions could be observed in BSA-docking mode, including cation-π (ARG472 and Arg458), anion-π (GLU399), alkyl-π (Arg196, His145, and Ala193), and alkyl (Ile455, Lys431, and Leu189). In the meantime, the MM/GBSA method was used to calculate the binding energies of the BSA and Lf proteins with the IR-1048 (**Table S1**). It was discovered that Lf had a higher docking binding energy (-10.83 kcal/mol) to the IR-1048 than BSA@IR-1048 (-9.74 kcal/mol), which explains why Lf has a greater effect on the enhancement of the luminescence of IR-1048 dye. It is possible to minimize the energy dissipation resulting from non-radiative leaps and efficiently limit the free torsion of IR-1048 with Lf. Furthermore, after a month of storage in PBS buffer, particle size of Lf@IR-1048 did not significantly alter (**Figure S10**). In conclusion, Lf protein exhibits stable and brilliant NIR-II optical characteristics, making it a superior choice for IR-1048 docking.

### NIR-II photothermal and photodynamic performances of protein@IR-1048 probes

**Figure 2:**
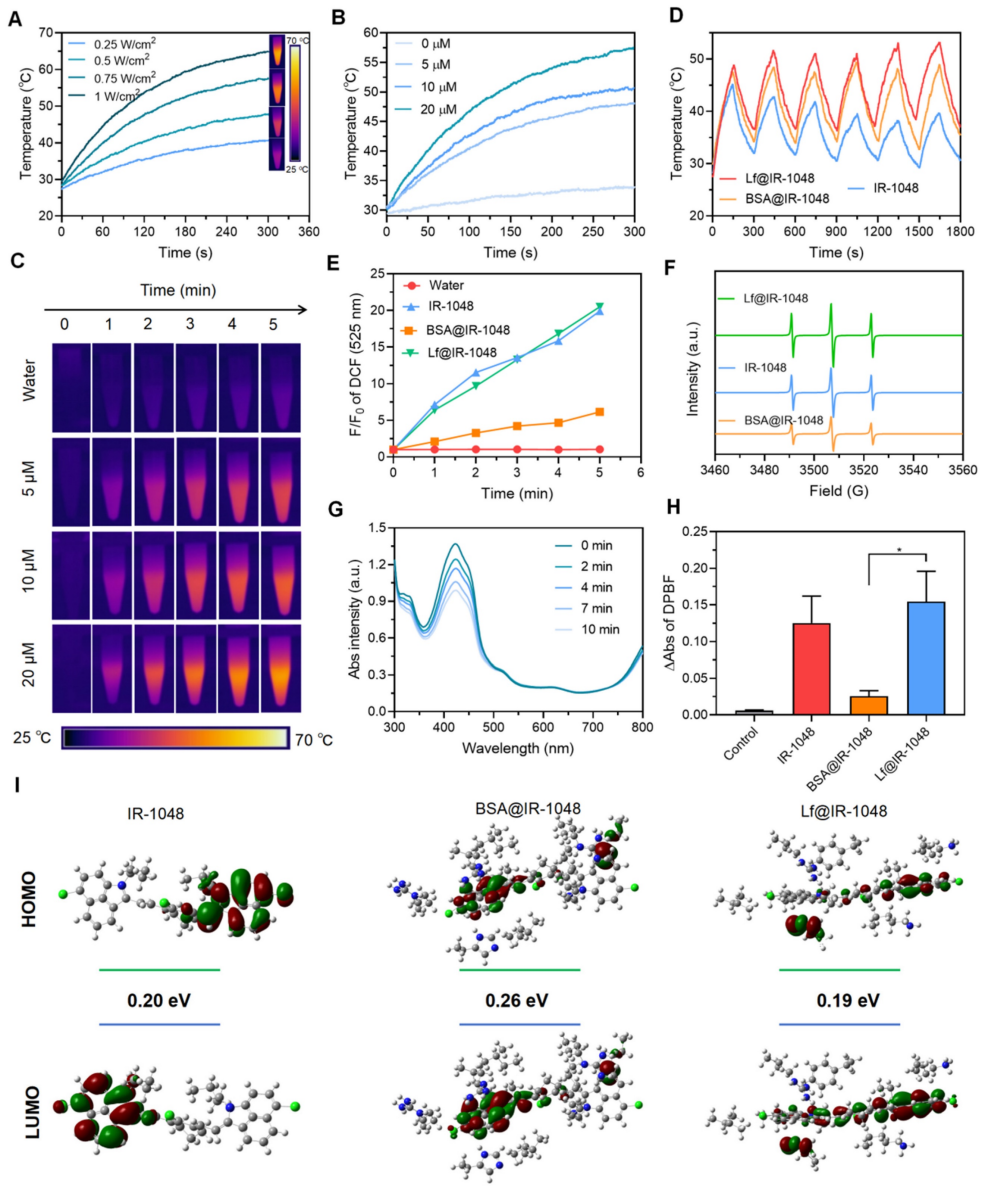
Photothermal heating curves of Lf@IR-1048 aqueous solutions at and different laser power densities **(A)** and different concentrations **(B)**. **(C)** Photothermal images of Lf@IR-1048 in solution with different concentrations under laser irradiation. **(D)** Temperature changes of Lf@IR-1048, BSA@IR-1048, and IR-1048 under six laser on/off cycles (1064 nm, 0.5 W/cm^2^). **(E)** Quantification of changes in fluorescence intensity from fluorescence spectra of DCFH-DA. **(F)** ESR spectra of ^1^O_2_ generated by IR-1048, BSA@IR-1048, and Lf@IR-1048. **(G)** The UV-vis spectra of DPBF treated with Lf@IR-1048 under 1064 nm laser irradiation (0.5 W/cm^2^) for 10 min. **(H)** Detection of generated ^1^O_2_ by IR-1048, BSA@IR-1048, and Lf@IR-1048 based on the absorption intensity of DPBF under a 1064 nm laser (0.5 W/cm^2^ for 10 min). **(I)** Highest occupied molecular orbital-lowest unoccupied molecular orbital (HOMO-LUMO) plots of IR-1048, Lf@IR-1048 and BSA@IR-1048 complexes simulated by density functional theory (the orbital energies shown in eV).

The NIR-II photothermal performance of every sample was investigated. An infrared camera was used to record the temperature changes of IR-1048, BSA@IR-1048, and Lf@IR-1048 at the same concentrations (10 μM) and laser intensity (0.5 W/cm^2^). While the temperature changes in IR-1048 and BSA@IR-1048 are smaller than those in Lf@IR-1048, the temperature of the Lf@IR-1048 clearly increased from 30.5 °C to 48.1 °C (**Figure S11**). Furthermore, by monitoring the temperature variations of IR-1048, BSA@IR-1048, and Lf@IR-1048 with and without the laser, the PTCEs of all probes were determined (**Figure S12**). **Figure S13** illustrated the correlation between the cooling time following laser irradiation and the negative natural logarithm of the temperature change. The PTCEs for IR-1048, BSA@IR-1048, and Lf@IR-1048, calculated using the standard technique in the Supplementary Information, are 30.3%, 33.5%, and 41.9%, respectively. The increased PTCE of Lf@IR-1048 results from its higher molar extinction coefficient. Next, it can be seen from the temperature variations of Lf@IR-1048 under various laser concentrations and intensities that the increase in temperature is dependent on both power and concentration (**Figure 2A** and **2B**). When compared to water alone, the accompanying thermal pictures demonstrated a notable rise in temperature for the Lf@IR-1048 at various concentrations when exposed to laser radiation (**Figure 2C**). Comparing to free IR-1048 molecule, the Lf@IR-1048 and BSA@IR-1048 exhibited remarkable photothermal stability, as demonstrated by the fact that their temperature remained relatively constant even after six cycles of cooling and irradiation (**Figure 2D**). Furthermore, during ten minutes of continuous laser irradiation, the spectra showed an unchanged absorption peak, which further verified the stability of the Lf@IR-1048 and BSA@IR-1048 (**Figure S14**). However, the absorption peak of IR-1048 underwent a weak decrease under continuous light, indicating the poor photostability of free IR-1048. Consequently, NIR-II PTT with stable photothermal conversion performance may be implemented using the Lf@IR-1048.

The ability of IR-1048, BSA@IR-1048, and Lf@IR-1048 to produce ROS in aqueous solution following 1064 nm (0.5 W/cm^2^) laser irradiation was then assessed, and its fluorescence enhancement could be utilized to evaluate the ROS level. This was done using a ROS fluorescent probe (DCFH-DA). IR-1048 and Lf@IR-1048 demonstrated a notable enhancement in DCF fluorescence when compared to the water sample and BSA@IR-1048 (**Figure S15**). DCF fluorescence was seen to be enhanced by almost 20 times in IR-1048 and Lf@IR-1048 (**Figure 2E**), indicating their effective ROS-generating capacity. Using ESR, the species of ROS in IR-1048 and Lf@IR-1048 under 1064 nm laser irradiation were identified. A common spin trapping agent for ^1^O_2_ in D_2_O is TEMP 39. The TEMPO-^1^O_2_ (TEMPO) spin adduct is characterized by an ESR signal of ^1^O_2_ in IR-1048, BSA@IR-1048, and Lf@IR-1048 solution under 1064 nm laser irradiation, as shown in **Figure 2F**, with a characteristic hyperfine splitting constant of 1:1:1 (a_N_ = 16.3 G). Lf@IR-1048 might be utilized for NIR-II PDT since the DPBF experiments shown that only the IR-1048 and Lf@IR-1048 were effective in producing ^1^O_2_ under 1064 nm laser (0.5 W/cm^2^) irradiation (**Figure 2G** and **2H**). To further analyze the type of reactive oxygen species, MB and DHR123 were used to detect hydroxyl radicals and superoxide radicals, respectively. There was no significant change in the absorption spectra of MB compared to the control group, indicating that no hydroxyl radicals were produced from the light-exposed Lf@IR-1048 solution (**Figure S16**). In addition, there was also no significant change in the fluorescence spectra of DHR123 compared to the control group, indicating that the light-exposed Lf@IR-1048 solution did not produce superoxide negative ions (**Figure S17**). To further understand the differential properties of ^1^O_2_ generation, we calculated the IR-1048, Lf@IR-1048 and BSA@IR-1048 at the B3LYP/6-31G (d) level using DFT. As shown in **Figure 2I**, the gap energy of the BSA@IR-1048 is 0.26 eV, which was higher than both IR-1048 (0.20 eV) and Lf@IR-1048 (0.19 eV). These results suggest that IR-1048 and Lf@IR-1048 are readily excited under energy, which may be related to the stronger generation of ^1^O_2_ under the same irradiation conditions. As a result, the Lf@IR-1048 probe has both excellent photothermal and photodynamic characteristics.

### NIR-II phototherapy of protein@IR-1048 at cellular level

**Figure 3:**
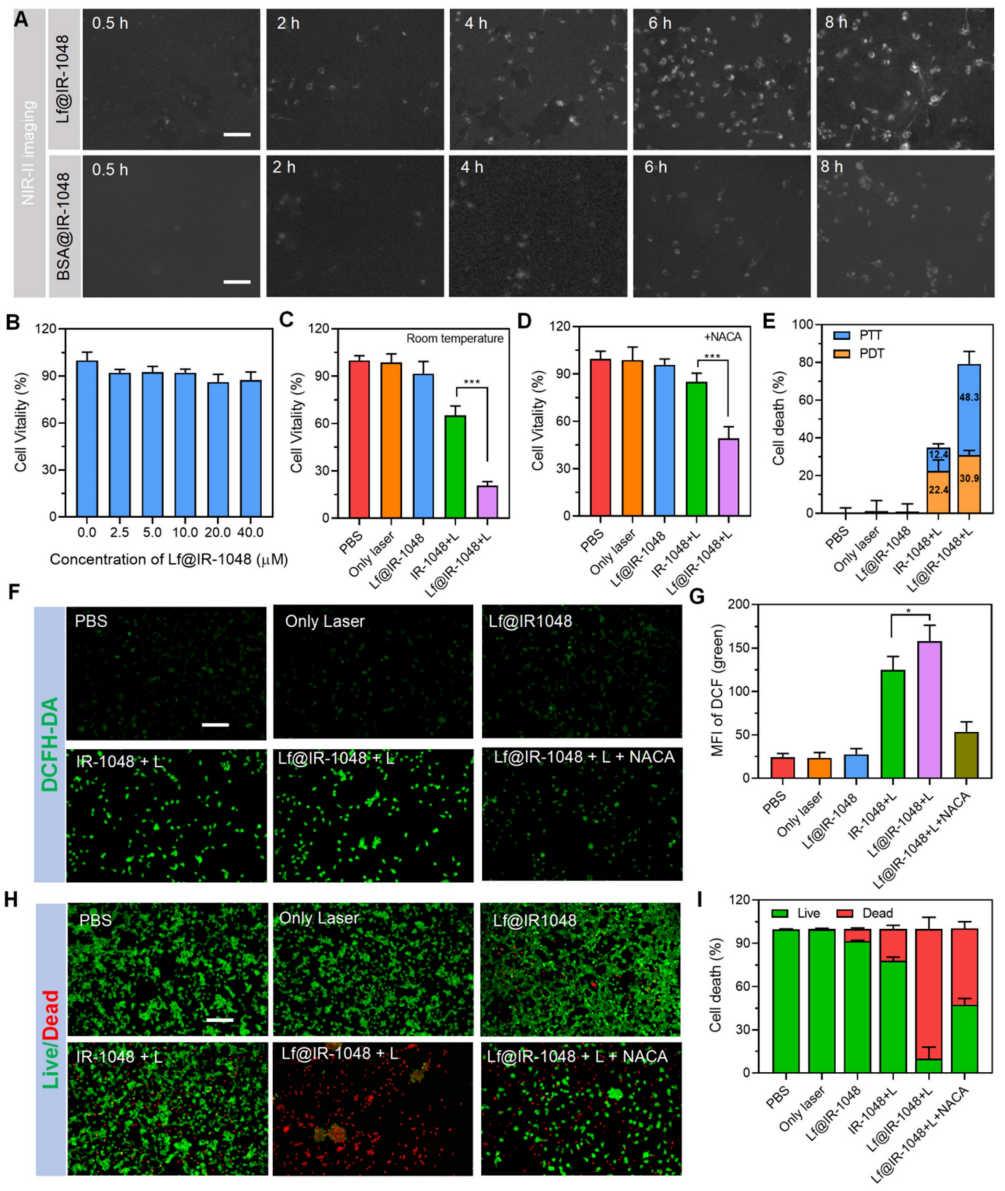
**NIR-II phototherapy of Protein@IR-1048 at cellular level**. **(A)** The NIR-II fluorescent images of CT26 cells incubated for different times in Lf@IR-1048 and BSA@IR-1048, respectively. **(B)** Cell viability of CT26 cells was assessed by CCK-8 assay after incubation with Lf@IR-1048 at various concentrations ranging from 0 to 40 μM. The viability of CT26 cells treated with different groups (PBS, Only laser, Lf@IR-1048, IR-1048 + Laser, and Lf@IR-1048 + Laser) at room temperature **(C)** and adding 1 mM NACA **(D)** under same concentration (20 μM) and laser intensity (0.5 W/cm^2^). **(E)** The ratio of dead cells induced by the PDT and PTT with different treatments (PBS, Only laser, Lf@IR-1048, IR-1048 + Laser, and Lf@IR-1048 + Laser). **(F)** The generation of ROS in the CT26 cells after administration with different groups (scale bar: 100 μm). **(G)** Quantification of the ROS in the CT26 cells from **(F)**. **(H)** The images of CT26 cells with Calcein AM/7-AAD costaining after different treatments (scale bar: 100 μm). **(I)** Quantification the proportion of live and dead cells from **(H)**. (All groups: PBS, Only laser, Lf@IR-1048, IR-1048 + Laser, Lf@IR-1048 + Laser, and Lf@IR-1048 + Laser +NACA). *P < 0.05 and ***P < 0.001.

Using an NIR-II fluorescent microscope (excitation: 980 nm and emission: 1048 nm), the endocytosis of nanoparticles was assessed prior to assessing the *in vitro* NIR-II PDT and PTT of protein@IR-1048. After varying the duration of their incubation, the BSA@IR-1048 and Lf@IR-1048 were incubated with CT26 cells and the fluorescence signal of cells was monitored. With longer co-incubation times, the NIR-II fluorescence increased, suggesting that CT26 cells may internalize protein@IR-1048 (**Figure 3A** and **S18**). Crucially, CT26 cells were able to endocytose Lf@IR-1048 more easily than BSA@IR-1048. This could be explained by the fact that cancer cells express the glycoprotein that transports iron 40. To verify the hypothesis that tumor cells uptake the Lf@IR-1048 *via* transferrin receptor (TfR), anti-Mouse CD71/TfR1 antibody (an inhibitor of TfR1) was applied. In this case, Lf was labeled with Cy3 for the subsequent study. The UV-vis and fluorescence spectra prove that the used product was Lf@Cy3 (**Figure S19**). Living cell imaging results for CT26 cells showed that the cellular uptake efficiency of the Lf@Cy3 was significantly suppressed in the presence of anti-TfR1, which was consistent with the inhibition of TfR1 expression (**Figure S20**). Moreover, the cellular uptake efficiency of the Lf@Cy3 in normal cells (Beas-2B cells) was lower than in tumor cells due to the low TfR1 expression in Beas-2B cells compared to tumor cells. All the above results indicated that the Lf complex could be uptaken by tumor cells through TfR1-mediated pathways.

Next, the conventional CCK-8 test was used to examine the possible cytotoxicity of Lf@IR-1048. After treating CT26 and Beas-2B cells with various doses of Lf@IR-1048 for 48 hours, only a minor cytotoxic effect was seen in **Figure 3B** and **S21**. In addition, the the normal cell viability was about 80.5% after treated with Lf@IR-1048 + Laser, while the viability of cancer cells was about 20.8% in same treatment (**Figure S22**), indicating that Lf@IR-1048 has good biosafety. Then, at ambient temperature, the therapeutic efficaciousness of Lf@IR-1048 mediated NIR-II PDT and PTT at the cell level were further established. Following treatment with IR-1048 + L and Lf@IR-1048 + L, the cell death rate was 34.8% and 79.2%, respectively, at room temperature (**Figure 3C**). On the other hand, we added reactive oxygen quencher (NACA) to each group and kept other treatment conditions constant, in order to analyze the contribution of PTT versus PDT, respectively. The cell mortality in the IR-1048 + L (12.4%) and Lf@IR-1048 + L (48.3%) groups was dramatically decreased when the culture medium was added NACA (**Figure 3D**). The NIR-II PDT and PTT were responsible for 30.9% and 48.3%, respectively, of cell death in the Lf@IR-1048 + L group (**Figure 3E**). By utilizing DCFH-DA, which can be oxidized by ROS to DCF showing green fluorescence, the production of ROS in CT26 cells was identified. Following treatment with PBS, laser, Lf@IR-1048, and Lf@IR-1048 + NACA, there was minimal ROS-associated green fluorescence in the cells (**Figure 3F**). On the other hand, ROS-specific green fluorescence was observed in both the IR-1048+ L and Lf@IR-1048 + L groups, suggesting that the formation of ROS was caused by the NIR-II laser when IR-1048 was present. More ROS in live cells was seen in the Lf@IR-1048 + L group (**Figure 3G**), which was attributed to enhanced hydrophilicity and targeted cell endocytosis.

Next, live/dead staining was employed to visually assess each treatment impact on cell death. As shown in **Figure 3H**, live cells (green staining) were observed almost exclusively in PBS, laser, and Lf@IR-1048 groups, and more than 90% of dead cells (stained red) in Lf@IR-1048 + L group demonstrated a significant contribution of cancer cellular deaths from the NIR-II PDT and PTT. After adding NACA to quench ROS, the percentage of dead cells was significantly reduced in Lf@IR-1048 + L group (**Figure 3I**). Then, the percentage of dead cells in each group was ascertained using flow cytometry in order to produce even more precise quantitative results. The percentages of dead cells for PBS, laser, Lf@IR-1048, IR-1048 + L, and Lf@IR-1048 + L were 0.2%, 0.1%, 9.1%, 27.8%, and 98.0%, respectively, in **Figure S23**. These findings thus offer strong evidence that NIR-II phototherapy with Lf@IR-1048 affects both PTT and PDT at the cellular level.

### Detection of DAMPs released from the dying cells

ICD causes dying cells that have been exposed to phototherapy to generate DAMPs, such as ATP, CRT, and HMGB1. These DAMPs send out "find-me" and "eat-me" signals that encourage the maturation of DCs, incite inflammation, and draw in immune cells 41-44. The ELISA kits were used to measure extracellular ATP, CRT, and HMGB1 under various treatment conditions. Extracellular ATP levels were higher following treatment with IR-1048 + L and Lf@IR-1048 + L groups compared to the PBS, laser, and Lf@IR-1048 groups (**Figure S24**). 67.6 nM of extracellular ATP was present in the Lf@IR-1048 + L group, while 49.5 nM was present in the IR-1048 + L group. Furthermore, CRT is a strong "eat-me" signal and a modulator of tumor immunogenicity.

**Figure 4:**
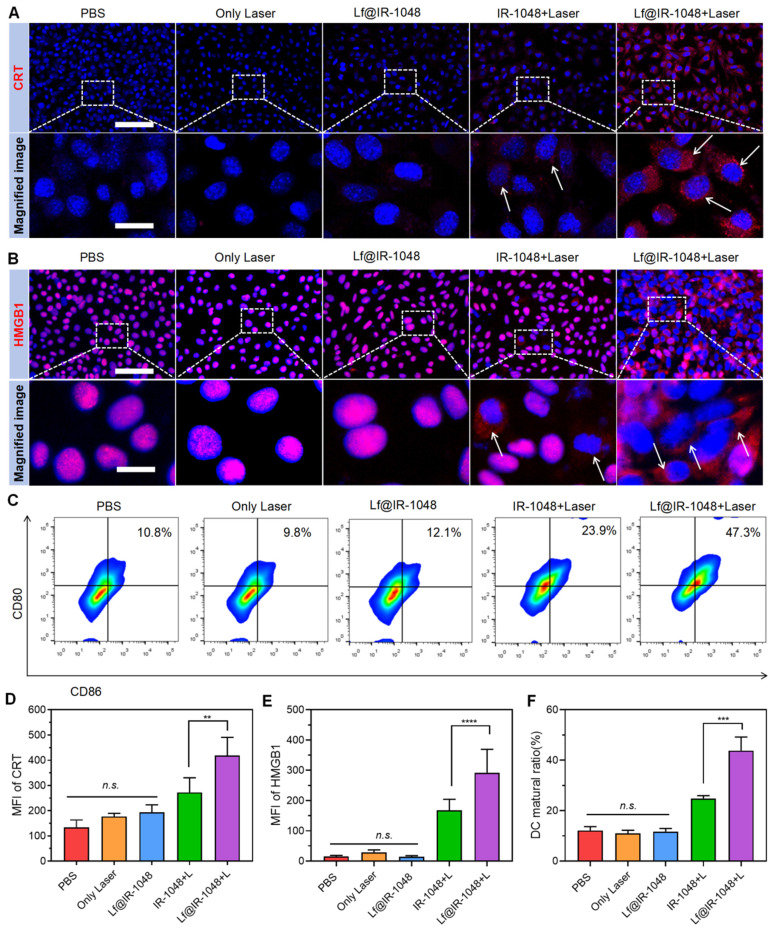
**(A)** Immunofluorescence images of CRT expression on the cell surface of CT26 cells treated with different groups. **(B)** Immunofluorescence staining determining the release of HMGB1 from the nucleus to the cytoplasm of CT26 cells in different treatment groups. **(C)** Flow cytometric data of DC maturation after treatment indifferent groups. **(D)** Quantification MFI of CRT expression. **(E)** Quantification MFI of HMGB1. **(F)** corresponding proportion of DC maturation. (All groups: PBS, Only laser, Lf@IR-1048, IR-1048 + Laser, and Lf@IR-1048 + Laser). *P < 0.05, **P < 0.01, and ***P < 0.001. *n.s.*, no significant difference between two groups.

The ELISA results, as indicated in **Figure S25**, showed that CRT treated with Lf@IR-1048 + L had the maximum release, indicating that NIR-II phototherapy is a useful tool for causing CRT exposure. Furthermore, compared to all other groups, Lf@IR-1048 + L produced a noticeably greater extracellular HMGB1 level (**Figure S26**). Next, using laser scanning confocal microscopy, the direct immunofluorescence images of CRT and HMGB1 in each group were assessed. Significant CRT expression (red fluorescence) was seen on CT26 cells treated with Lf@IR-1048 following irradiation, as seen in **Figure 4A**. In contrast, the PBS, laser, and Lf@IR-1048 groups showed almost any indication of cell surface CRT expression. As far as we know, during ICD, HMGB1 is released from the nucleus of dying cells. The translocation of HMGB1 from the nucleus to the extracellular space was shown to be greatly increased by Lf@IR-1048 + L treatment (**Figure 4B**). Immune signals such as CRT, ATP, and HMGB1 can effectively up-regulate the expression of CD80 and CD86 signals in dc within a certain concentration range and stimulate DC maturation 45. To detect the maturity of DCs, BMDCs were treated with in different groups and stained with CD11c, CD80, and CD86 antibodies. The highest expression of CD80 and CD86 in Lf@IR-1048 + Laser was 47.3%, which was 4.38 fold that of the PBS group (**Figure 4C**). The corresponding quantification of CRT expression, HMGB1 release, and DC maturation were significantly increased in the Lf@IR-1048 + Laser group (**Figure 4D-4F**). Thus, it appears that Lf@IR-1048 promotes ICD following irradiation based on the observation of increased ATP and HMGB1 levels as well as up-regulation of CRT expression.

### Multimodal imaging of Lf@IR-1048 *in vivo*

Real-time monitoring of efficacious treatment is contingent upon the availability of potential diagnostic imaging advice. Animal NIR-II fluorescence imaging has been extensively studied due to its low autofluorescence and low photon scattering. Prior to examining the *in vivo* multimodal imaging characteristics of the Lf@IR-1048 probe, a hemolysis assay was used to assess its biosecurity. When erythrocytes were co-cultured with varying concentrations of Lf@IR-1048 probe, no discernible hemolysis was seen (**Figure S27**). Balb/c mice (n = 3) were given intravenously 2 mg/kg of Lf@IR-1048 to further evaluate the pharmacokinetics of the drug. Blood samples were taken at 0.5, 1, 2, 4, 8, 12, 24, and 36 hours after the injection. Protein protection was found to be responsible for the long-term cycling property of IR-1048 molecules *in vivo*, according to fluorescence examination of blood samples (**Figure S28**).

**Figure 5:**
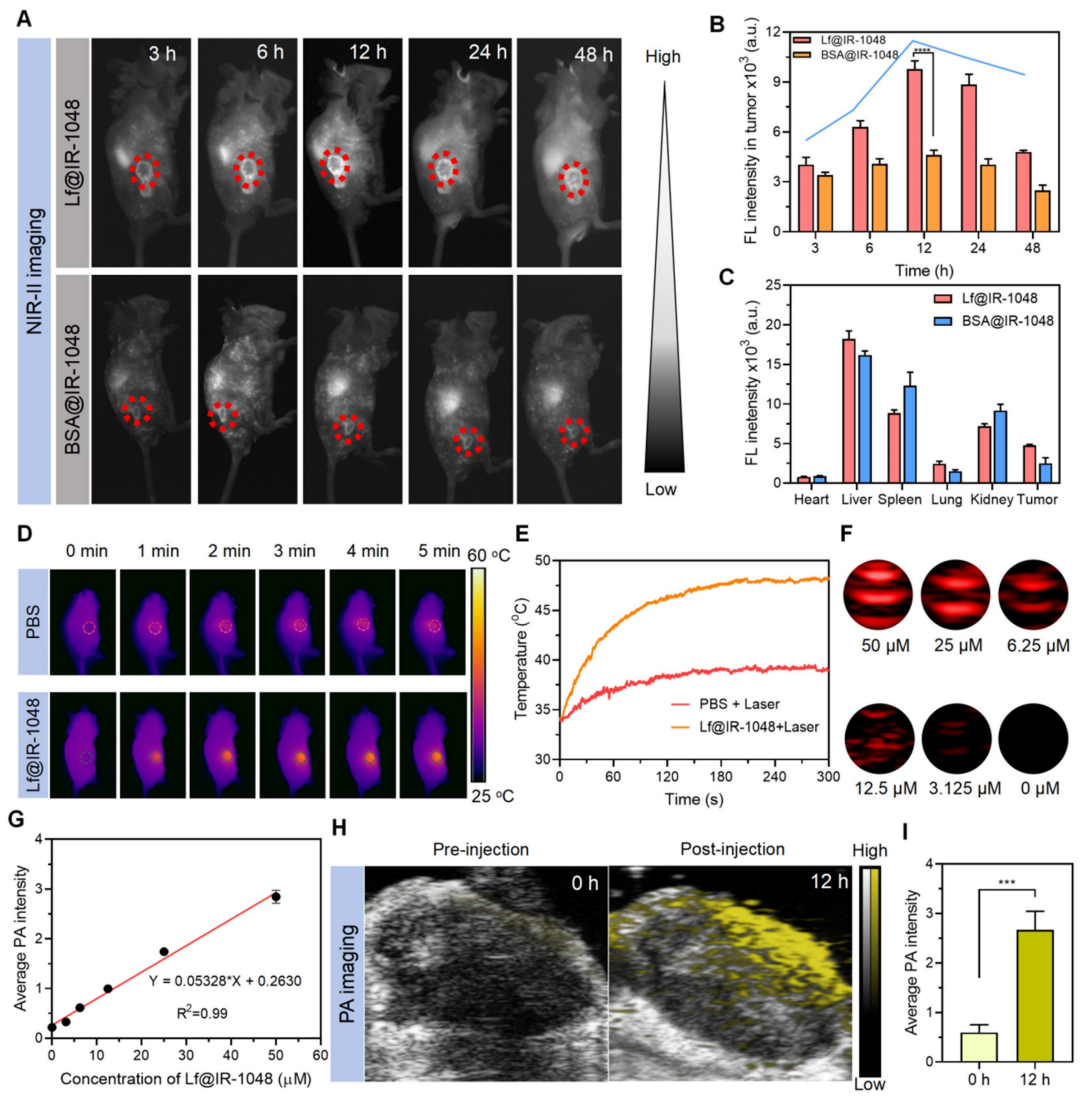
**Multimodal imaging of Lf@IR-1048* in vivo****.* **(A)** Time-dependent *in vivo* NIR-II fluorescence imaging of CT26 tumor-bearing mice after the intravenous injection of BSA@IR-1048 and Lf@IR-1048 (IR-1048: 2 mg/kg) under excitation wavelength of 980 nm. **(B)** Corresponding fluorescence intensity of tumor sites at different time-points. **(C)** Fluorescence intensity in major organs and tumors at 48 h post-injection with BSA@IR-1048 and Lf@IR-1048. **(D)** *In vivo* photothermal imaging of CT26 tumor-bearing mice after intravenous administration of Lf@IR-1048. **(E)** Temperature changes at tumor sites with administration of PBS and Lf@IR-1048 (IR-1048: 2 mg/kg) after laser irradiation. **(F)** PA images of Lf@IR-1048 with different concentrations (50, 25, 6.25, 12.5, 3.125, and 0 μM). **(G)** PA intensity *versus* the concentration of Lf@IR-1048. **(H)** *In vivo* PA images of CT26 tumor-bearing mice before and after intravenous injection of Lf@IR-1048 (IR-1048: 2 mg/kg). **(I)** Corresponding PA intensity of tumor sites from **(H)**. ***P < 0.001. *n.s.*, no significant difference between two groups.

The* in vivo* NIR-II fluorescence imaging of tumors was next examined by intravenously injecting BSA@IR-1048 and Lf@IR-1048 probes (IR-1048: 2 mg/kg) into the CT26 tumor-bearing mice. The NIR-II fluorescence signal in the tumor was clearly apparent in the Lf@IR-1048 group at 6 hours after injection, and it peaked at around 12 hours later (**Figure 5A** and **5B**). Nevertheless, within 24 hours of injection, the tumors in the BSA@IR-1048 group show an unremarkable shift in the NIR-II fluorescence signal. Thus, the Lf@IR-1048 probe has better tumor targeting than the BSA@IR-1048 probe. To further evaluate the probe capabilities, tumors and organs of mice from the BSA@IR-1048 and Lf@IR-1048 groups were taken 48 hours after injection and subjected to *ex vivo* NIR-II fluorescence imaging (**Figure S29**). Liver, spleen, kidneys, and tumors all displayed high NIR-II fluorescence intensity for Lf@IR-1048, while the liver, spleen, and kidneys displayed high NIR-II fluorescence signal for BSA@IR-1048 (**Figure 5C**). The large amount of NIR-II fluorescent signal in the spleen is due to protein carriers that can be ingested by the spleen 46.

Additionally, the foundation of photothermal imaging (PTI) is the high NIR-II PTCE and tumor targeting of Lf@IR-1048. Animals having CT26 tumors were given intravenous injections of PBS and Lf@IR-1048. Twelve hours later, the animals were exposed to a 1064 nm laser (0.5 W/cm^2^). The tumor in the Lf@IR-1048 group warmed up from 34.2 °C to 48.3 °C, as seen in **Figure 5D** and **5E**. In contrast, the tumors treated with PBS only saw a 4.9 °C temperature increase under the same laser irradiation, indicating a possible involvement for Lf@IR-1048 in the NIR-II PTT impact on tumors. Furthermore, Lf@IR-1048 is anticipated to be imaged by the PA approach, which benefits from the red-shifted absorption peak following protein-hitchhiking of IR-1048 molecules. First, Lf@IR-1048 signal intensity and *in vitro* PA images were assessed in solution at various doses (**Figure 5F**). **Figure 5G** demonstrated a strong linear correlation between the PA signal intensity and the concentration of Lf@IR-1048. Intravenous injection of Lf@IR-1048 was used for* in vivo* PA imaging of mice with CT26 tumors, both before and after 12 hours post-injection (**Figure 5H**). Twelve hours after the injection, the PA signal at the tumor site dramatically increased, and semi-quantitative evaluations of the PA signal intensity revealed variations in intensity both before and after that time (**Figure 5I**). The multimodal imaging feature of Lf@IR-1048 probe allows for real-time drug accumulation monitoring in tumors, clinical treatment time and dose calculations, and efficient real-time diagnosis and therapy.

### Anti-tumor efficacy of Lf@IR-1048 *in vivo*

**Figure 6:**
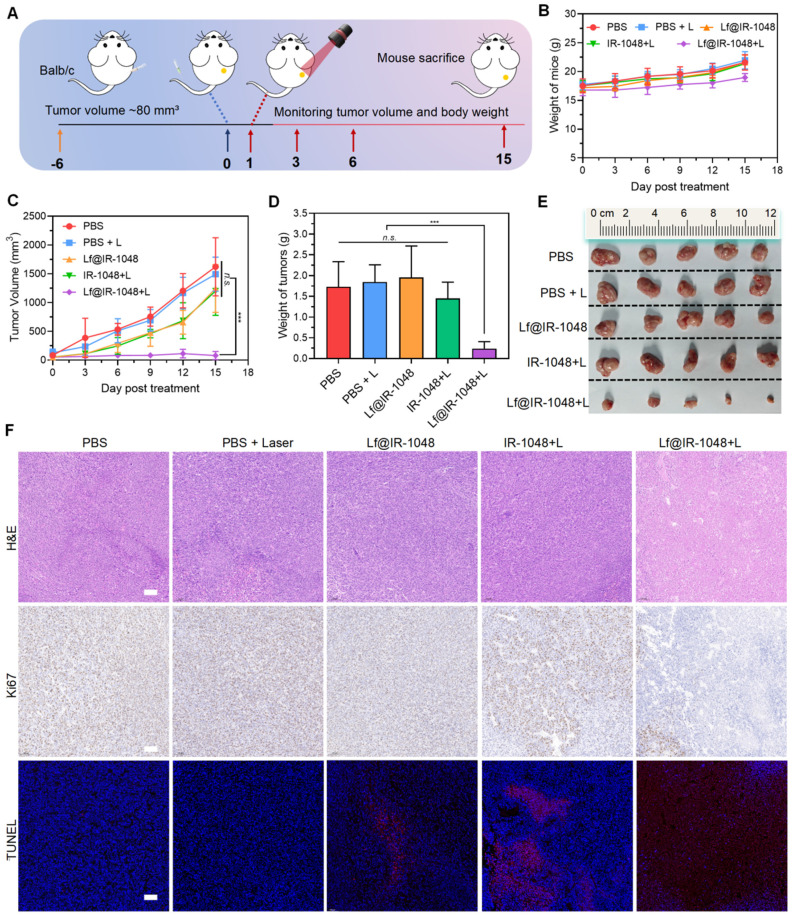
**Anti-tumor efficacy of Lf@IR-1048* in vivo.* (A)** Schematic illustration for CT26 tumor treatment. Body weight **(B)** and tumor growth curve **(C)** of mice with various treatments including PBS, PBS + L, Lf@IR-1048, IR-1048 + L, and Lf@IR-1048 + L (IR-1048: 2 mg/kg). **(D)** Mean tumor weight of each group after 15 day of treatment. **(E)** Photograph of tumors stripped from mice at the end of treatment. **(F)** H&E, Ki67, and TUNEL stained tumor sections of each group. Scale bar = 100 μm, ***p < 0.001. *n.s.*, no significant difference between two groups.

We also investigated Lf@IR-1048's anticancer efficacy for NIR-II PDT and PTT *in vivo*. Mice having a CT26 tumor (~80 mm^3^) were injected intravenously with PBS, Lf@IR-1048, and IR-1048 (IR-1048: 2 mg/kg). These mice were then split into five groups (n = 5): PBS, PBS + L, Lf@IR-1048, IR-1048 + L, and Lf@IR-1048 + L. **Figure 6A** illustrates this process. The tumors were exposed to laser irradiation (0.5 W/cm^2^, 10 min) at 12 h following injections of PBS, IR-1048, and Lf@IR-1048, respectively. Throughout therapy, mice in each group had their body weight and tumor volume measured every three days. The body weight fluctuations of all the mice exhibited a rising trend (**Figure 6B**), indicating that the Lf@IR-1048 injection dose had very little negative effect on the mice. The CT26 tumor growth was not inhibited by the PBS, PBS + L, Lf@IR-1048, or IR-1048 + L groups, as **Figure 6C** shows. In contrast to the PBS group, the combined effects of Lf@IR-1048 with NIR-II laser on tumor development were 94.8% inhibitory, suggesting a desired therapeutic impact from NIR-II PDT and PTT.

Simultaneously, the best inhibitory impact on tumor growth was confirmed by weight (**Figure 6D**) and photos (**Figure 6E**) of the excised tumors from the mice in each group that were euthanized on the fifteenth day following the initial therapy. Resected tumors were sectioned and stained with H&E, Ki67, and TUNEL in order to evaluate the effectiveness even more (**Figure 6F**). According to the tumor growth statistics, the tumors in the Lf@IR-1048 + L therapy group had significant necrosis when stained with H&E. Furthermore, Ki67 antibody staining unequivocally showed that the Lf@IR-1048 + L-treated group had the greatest reduction of cell proliferation, confirming the high effectiveness of PDT and PTT-mediated treatment of Lf@IR-1048. Red fluorescence TUNEL staining further revealed that Lf@IR-1048 + L had a notably higher degree of tumor cell necrosis than the other groups. Thus, NIR-II phototherapy mediated by Lf@IR-1048 demonstrated significant growth inhibition of tumors.

**Figure 7:**
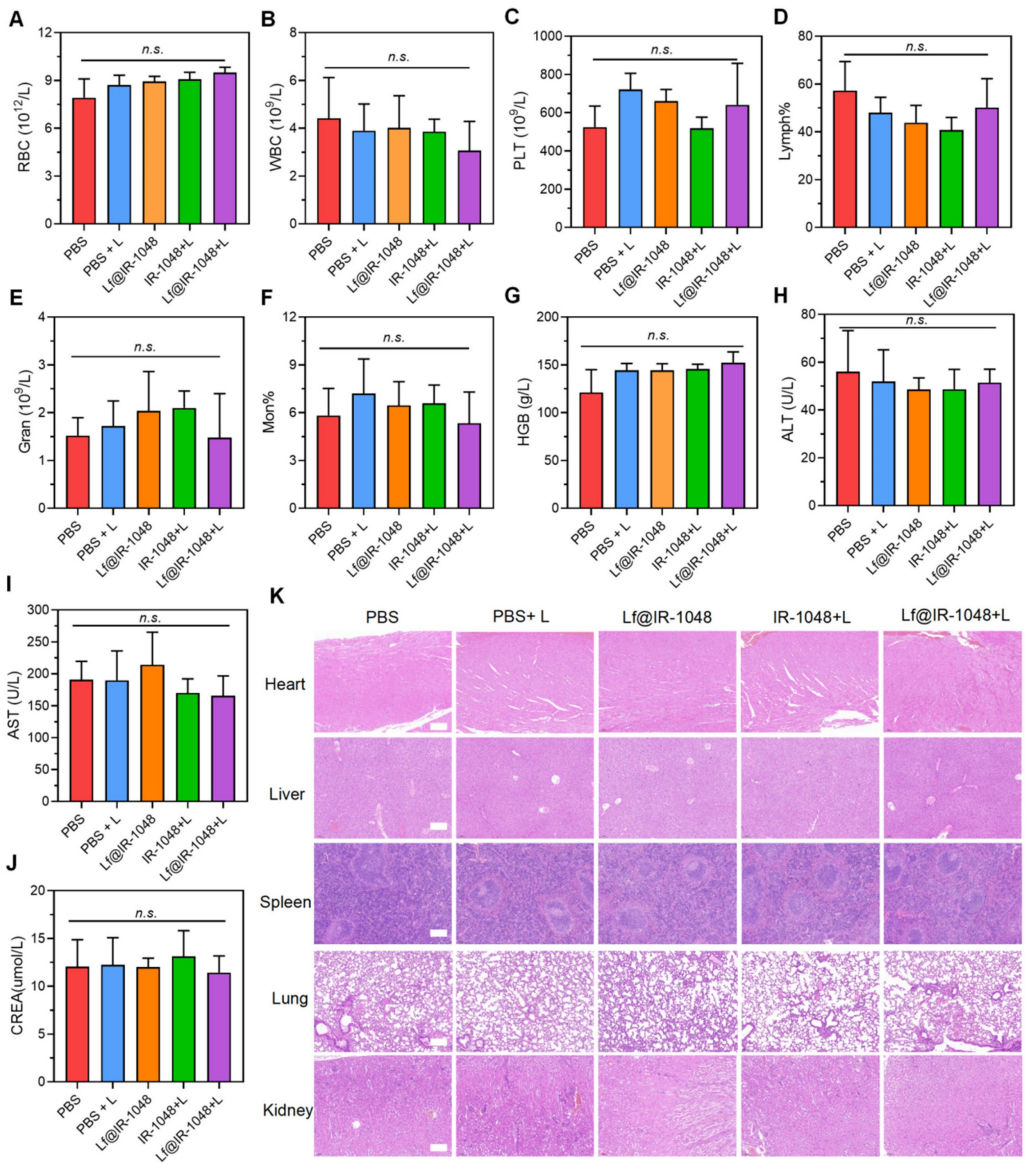
** The biosafety of Lf@IR-1048 (IR-1048: 2 mg/kg) based NIR-II phototherapy. (A)**-**(G)** Blood routine and **(H)**-**(J)** blood biochemical analysis (n = 5) of mice in each group. **(K)** H&E staining of heart, liver, spleen, lung, and kidney of mice in each group of PBS, PBS + L, Lf@IR-1048, IR-1048 + L, and Lf@IR-1048 + L. (Scale bar = 200 μm). *n.s.*, no significant difference between two groups.

At the conclusion of therapy, blood samples were taken for blood analysis from mice in each group in order to further explore the biosafety of Lf@IR-1048 based NIR-II phototherapy. In experimental mice with or without laser irradiation, the effects of injection with Lf@IR-1048 on erythrocytes, leukocytes, platelets, lymphocytes hemoglobin, and mean erythrocyte hemoglobin concentration did not exhibit significant differences when compared with the PBS group (**Figure 7A-7G**). The normal range was seen in the routine blood test readings of the aforementioned groups. Important blood chemistries including AST and ALT did not exhibit aberrant fluctuations across all groups (**Figure 7H** and **7I**), suggesting that liver function was not impaired by Lf@IR-1048. The other blood chemistry parameter for CREA in each group was found to be within the normal range (**Figure 7J**), indicating that Lf@IR-1048 does not appear to be affecting renal function. Furthermore, each group of mice had its major organs (heart, liver, spleen, lungs, and kidneys) stained with H&E for additional biosafety assessment. Following various treatments, all H&E staining images revealed no appreciable adverse effects in the main organs (**Figure 7K**). According to these findings, Lf@IR-1048 is anticipated to be a safe, effective phototherapy agent for NIR-II PDT and PTT, as well as for preventing the growth of tumors.

### Anti-tumor photo-immunotherapy of Lf@IR-1048 *in vivo*

**Figure 8:**
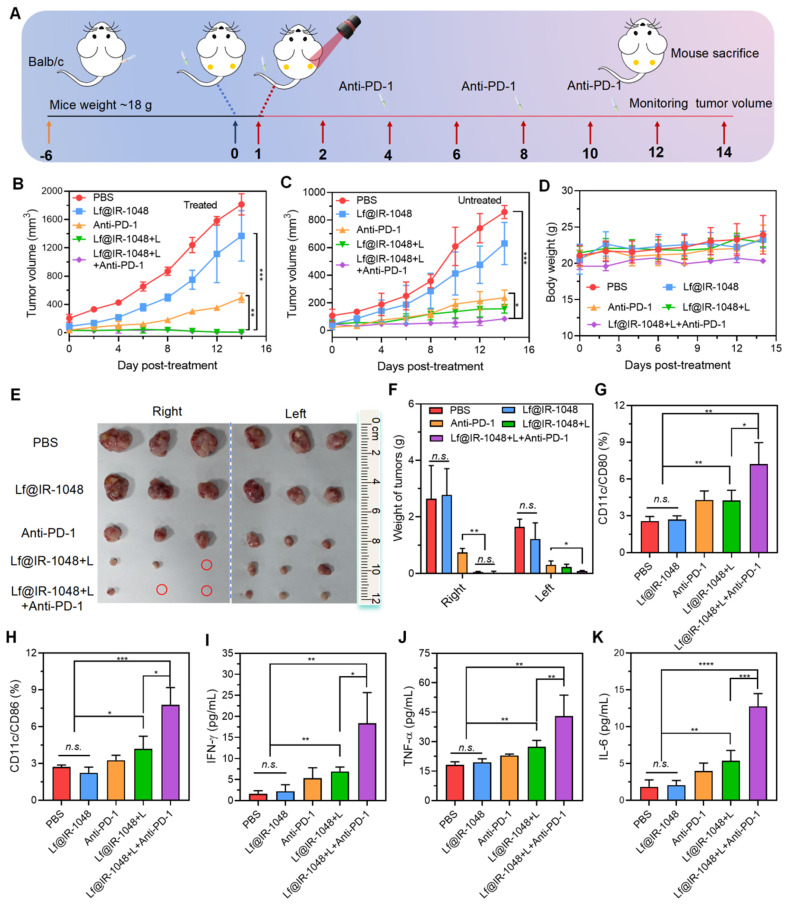
** Anti-tumor photo-immunotherapy of Lf@IR-1048* in vivo****.* **(A)** Schematic illustration of bilateral CT26 tumor model for photo-immunotherapy. **(B)** Right-sided tumor growth curves of mice with different treatments including PBS, Lf@IR-1048, Anti-PD-1, Lf@IR-1048 + L, and Lf@IR-1048 + L + anti-PD-1 (IR-1048: 2 mg/kg, anti-PD-1: 20 μg). **(C)** Left-sided tumor growth curves of mice without laser irradiation. **(D)** Body weight curves of mice in each groups. **(E)** Photograph of right- and left-sided tumors stripped from mice at the end of treatment. **(F)** Right- and left-sided tumor weight of each group after 14 day of treatment. Percentage of DCs maturation with CD86 **(G)** and CD80 **(H)** in DCs from tumor-draining lymph nodes induced by different treatment on mice. **(I)**-**(K)** Cytokine levels of IFN-γ, TNF-α, and IL-6 in mouse serum. Data are shown as mean ± standard deviation *p < 0.05, **p < 0.01, ***p < 0.001, and ****p < 0.0001. *n.s.*, no significant difference between two groups.

In order to study the systemic immune response brought about by Lf@IR-1048-mediated photo-immunotherapy, we employed a bilateral tumor model, wherein mice were subcutaneously injected with CT26 cells. **Figure 8A** showed that the left-sided tumor, which was not exposed to laser light, was identified as the distant abscopal tumor, whereas the right-sided tumor was designated as the primary tumor. At the end of the treatment period, the volume of the primary tumors was significantly lower in all mice treated with Lf@IR-1048-mediated photo-immunotherapy or phototherapy than in the PBS, Lf@IR-1048, and anti-PD-l groups (**Figure 8B**). However the distal tumor volume in Lf@IR-1048-mediated phototherapy mice showed a greater growth rate (**Figure 8C**), indicating that the distal advantage of phototherapy without anti-PD-l was limited. The distal tumor development was effectively suppressed following Lf@IR-1048-mediated photo-immunotherapy. The body weight fluctuations of all the mice exhibited a rising trend (**Figure 8D**), indicating that the Lf@IR-1048 based photo-immunotherapy had very little negative effect on the mice.

Furthermore, the strongest inhibitory impact on tumor growth was established by pictures (**Figure 8E**) and weight (**Figure 8F**) of the bilateral tumors from the deceased mice in each group on the fourteenth day following the first treatment. Thereafter, we employed flow cytometry to measure the levels of DC maturation in tumor-draining lymph nodes and ELISA to detect serum inflammatory cytokine levels in order to confirm whether Lf@IR-1048-mediated photo-immunotherapy could encourage DCs maturation and secretion of immune cytokines. Anticipating this, photo-immunotherapy mediated by Lf@IR-1048 induced a greater rate of DCs maturation (17.5%) in contrast to the other groups (**Figure 8G** and **8H**). When combined, these results imply that DCs maturation can be efficiently accelerated by laser elimination of tumor-associated antigens in tumors containing anti-PD-l. Following various treatments, CT26 tumor-bearing mice frequently had higher serum inflammatory factors (IFN-γ, TNF-α, and IL-6). Among all the groups, the Lf@IR-1048-mediated photo-immunotherapy cytokine production was the highest (**Figure 8I-8K**), indicating that this modality has a role in initiating immunological responses. These outcomes confirmed that photo-immunotherapy mediated by Lf@IR-1048 may successfully suppress tumor development and elicit an immune response.

**Figure 9:**
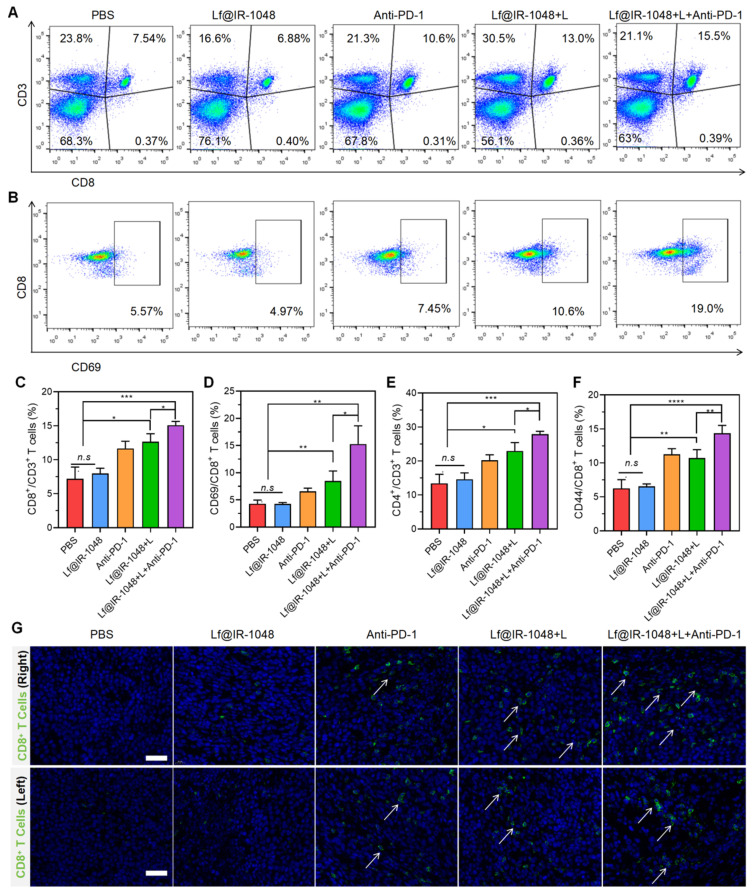
**(A)** The percentage of CD8^+^ T cells in all leukocytes with CD3 expression stimulated with different treatments and measured by flow cytometry. **(B)** The percentage of activation CD8^+^ T cells with the expression of CD69 after different treatments. Quantitative analysis of the percentage of anti-tumor CD8^+^ **(C)** and CD4^+^ T **(D)** cells among leukocytes in different groups. Quantitative analysis of the percentage of activation CD8^+^ T cells with the expression of CD69 **(E)** and memory CD8^+^ T cells with expression of CD44 **(F)** in different groups. **(G)** Immunofluorescence staining images of tumor-infiltrating CD8^+^ T cells (green fluorescence) in bilateral CT26 tumors after treated with different groups (scale bar: 40 μm), including PBS, Lf@IR-1048, Anti-PD-1, Lf@IR-1048 + L, and Lf@IR-1048 + L + anti-PD-1. *p < 0.05, **p < 0.005, ***p < 0.001, and ****p < 0.0001. *n.s.*, no significant difference between two groups.

We also looked at Lf@IR-1048's capacity to stimulate T cell immune response* in vivo* when combined with anti-PD-1 under laser irradiation, which is crucial for the removal of tumor cells. After dissecting to extract primary tumor draining lymph nodes, the percentage of cytotoxic T lymphocytes (CD8^+^ and CD4^+^ T cells) in CT26-bearing mice treated in various groups was measured using flow cytometry on the fourteenth day. In comparison to Lf@IR-1048 + L (13.0%) and anti-PD-1 (10.6%), the Lf@IR-1048 + L + anti-PD-1 group (15.5%) had a greater percentage of CD8^+^ T cells, as seen in **Figure 9A** and **9C**, indicating stronger anti-tumor immunity. Furthermore, an improved anti-tumor immunity was suggested by the higher percentage of CD4^+^ T cells in the Lf@IR-1048 + L + anti-PD-1 group (**Figure S30** and **9D**). In addition, T cell activation and immunological memory were strongly promoted by Lf@IR-1048 + L + anti-PD-1 vaccination, as shown by increased CD69 expression on T cells (**Figures 9B** and **9E**) and a greater proportion of memory CD44^+^ T cells (**Figure S31** and **9F**). The immunofluorescence staining images of the bilateral tumors also demonstrated a noticeable increase in CD8^+^ T cells (green fluorescence) following treatment with Lf@IR-1048 + L + anti-PD-1 (**Figure 9G**). This suggests that the tumors have successfully been invaded by cytotoxic T lymphocytes following photoimmunotherapy. It is fair to assume that the Lf@IR-1048 photo-immunotherapy could produce an adaptive immune response against the growth of distant cancers based on the findings mentioned above.

## Conclusions

The biological applications of a planar NIR-II cyanine dye are hindered by its strong π-π stacking in aqueous solution, which forms H-aggregates with the spectral blue shifts and fluorescence quenching. Lf was selected as the docking protein to load the IR-1048 molecule and create the Lf@IR-1048 probe, which showed NIR-II fluorescence that was "ON" and a red-shifted absorbance peak. Furthermore, it was shown by *in vitro* and* in vivo* experiments that the Lf@IR-1048, which has a superior targeting of tumor cells, can effectively produce ^1^O_2_ and transform photons into heat energy using one laser source. The* in vivo* multimodal imaging properties demonstrated efficient tumor growth inhibition with the Lf@IR-1048 mediated NIR-II phototherapy under real-time monitoring. Additionally, in bilateral CT26 tumor-bearing mice, Lf@IR-1048-based photo-immunotherapy shows more significant anti-tumor activities, accompanied by the release of DAMPs from dying cancer cells to encourage the maturation of DCs and the infiltration of cytotoxic T lymphocytes. As a result, Lf@IR-1048 showed significant promise as a viable and secure photo-immunotherapy approach for malignancies.

## Supplementary Material

Supplementary figures and calculations.
