# Supplementary material for: Lactoferrin docking NIR-II cyanine dye as a potentiated phototheranostic for synchronous multimodal bioimaging and tumor photo-immunotherapy
Source: Theranostics. 2024 Oct 14;14(17):6671–91. doi: 10.7150/thno.102663 (PMC11519796; doi:10.7150/thno.102663)
Supplement: Supplementary file 1 — Supplementary figures and calculations. [file thnov14p6671s1.pdf]

Supplementary material

Lactoferrin docking NIR-II cyanine dye as a potentiated phototheranostic for synchronous multimodal bioimaging and tumor photo-immunotherapy

Lifeng Hang,[1#] Haijian Li,[1#] Meng Li,[1#] Yiqiang Sun,[2] Wenjiao Wu,[1] Laiping Fang,[1] Yanzhao Diao,[3] Hong Qu,[3] Tao Zhang,[4] Shumei Li,[1*] Guihua Jiang[1,3*]*

[1] The Department of Medical Imaging, Guangzhou Key Laboratory of Molecular Functional Imaging and Artificial Intelligence for Major Brain Diseases, The Affiliated Guangdong Second Provincial General Hospital of Jinan University, Guangzhou 518037, P. R. China

[2] School of Chemistry and Chemical Engineering, University of Jinan, Jinan 250022, P. R. China

[3] School of Medicine, Jinan University, Guangzhou, 510632, P. R. China

[4] School of Physical and Mathematical sciences, Nanyang Technological University, 21 Nanyang Link, Singapore, 637371, Singapore

[#]These authors contributed equally to this work.

*Corresponding authors:

E-mail addresses: chm_sunyq@ujn.edu.cn (Yiqiang Sun); shumeili0412@qq.com (Shumei Li); jianggh@gd2h.org.cn (Guihua Jiang)

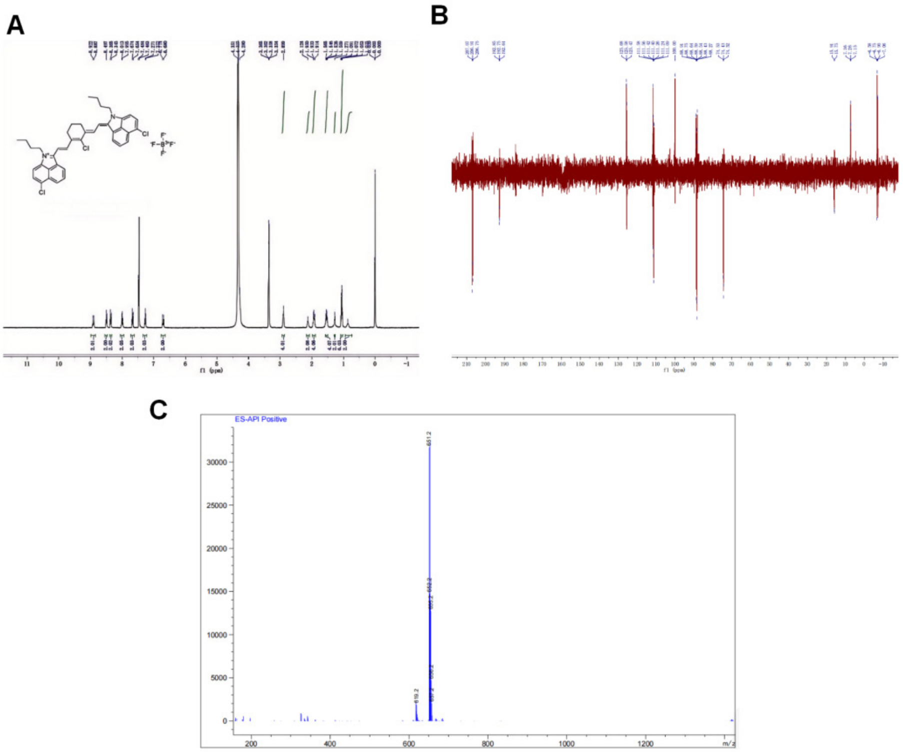

Figure S1. The ^{1}H NMR (A), ^{13}C NMR (B), and EI-MS (C) of IR-1048.

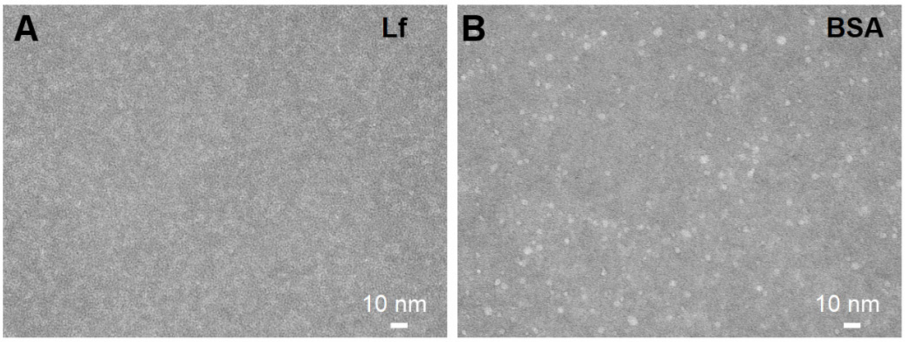

Figure S2. TEM images of Lf and BSA.

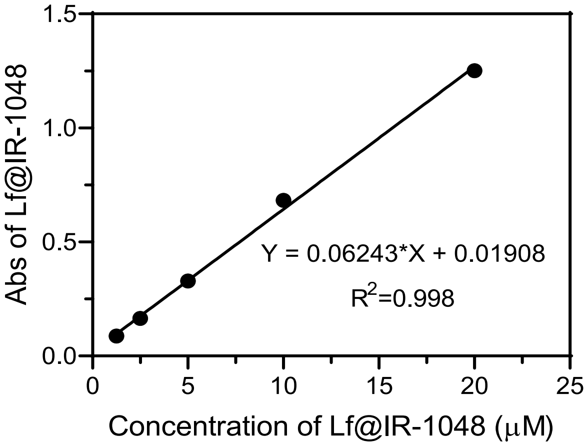

Figure S3. The linear relationship between the concentration and maximum absorbance of Lf@IR-1048 probes.

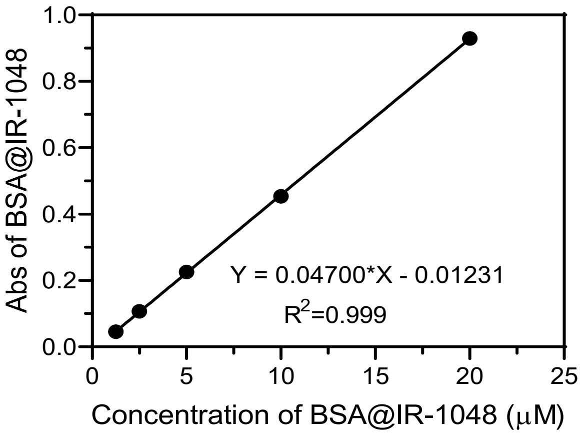

Figure S4. The linear relationship between the concentration and maximum absorbance of BSA@IR-1048 probes.

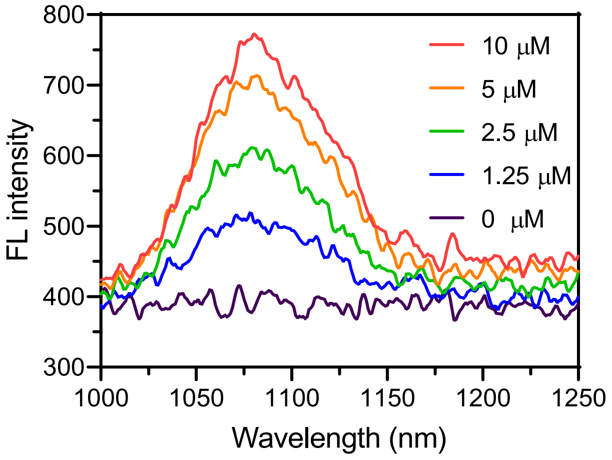

Figure S5. Fluorescence spectra of Lf@IR-1048 with different concentration in water under excitation wavelength of 980 nm.

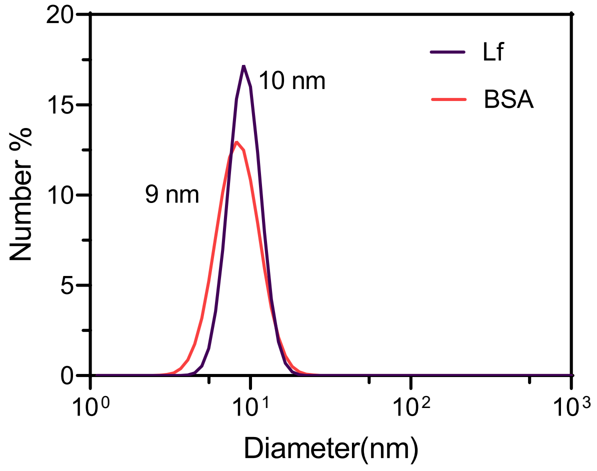

Figure S6. The hydrodynamic sizes of BSA and Lf.

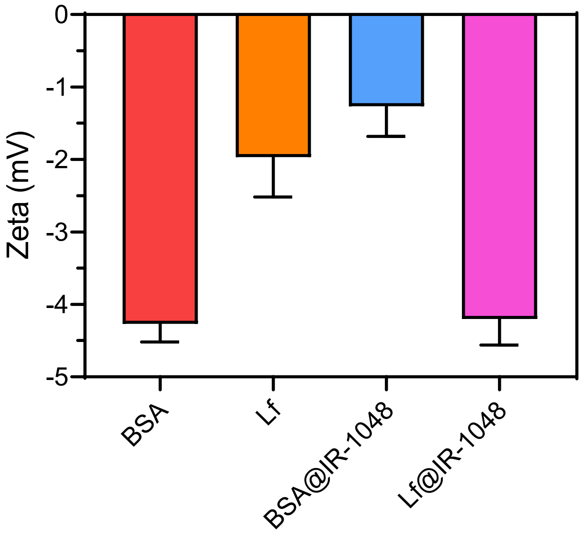

Figure S7. Zeta potentials of BSA, Lf, BSA@IR-1048, and Lf@IR-1048.

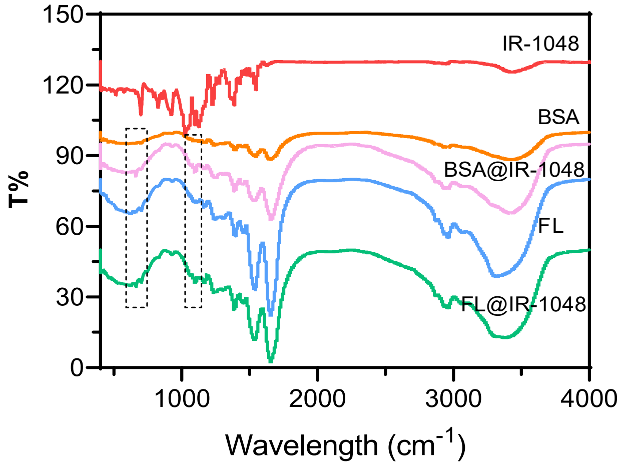

Figure S8. Fourier infrared spectra of IR-1048, BSA, Lf, BSA@IR-1048, and Lf@IR-1048.

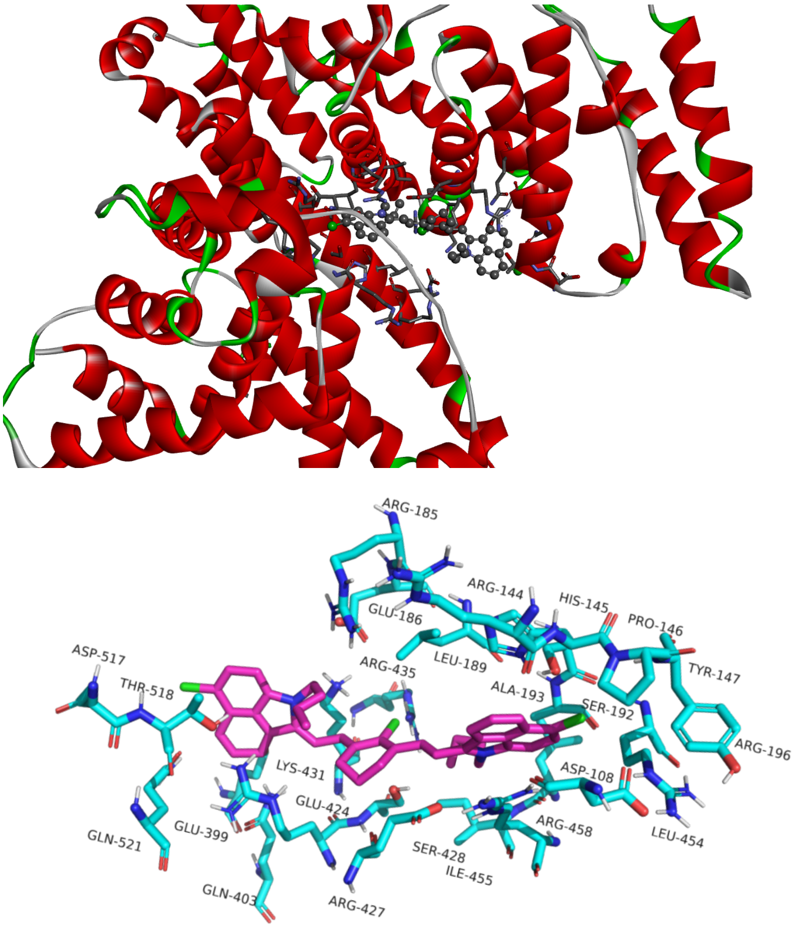

Figure S9. Theoretical simulations of IR-1048 docking to BSA protein in the gliding docking mode.

Table S1. Top 10 binding energies (kcal/mol) of IR-1048 in docked conformations with Lf and BSA proteins.

Docking configurations	Lf@IR-1048	BSA@IR-1048
1	-7.47	-7.89
2	-10.83	-7.78
3	-7.82	-8.43
4	-7.62	-7.28
5	-9.39	-7.94
6	-10.69	-9.74
7	-7.03	-6.15
8	-8.55	-8.92
9	-7.89	-8.71
10	-8.05	-7.42

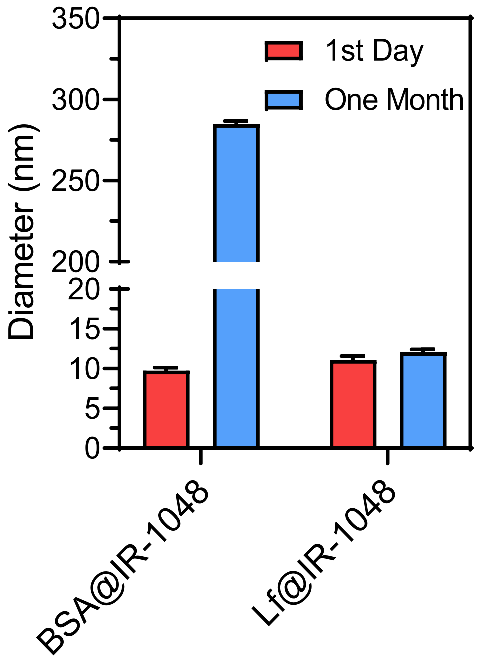

Figure S10. The hydrodynamic sizes of BSA@IR-1048 and Lf@IR-1048 in PBS for one month.

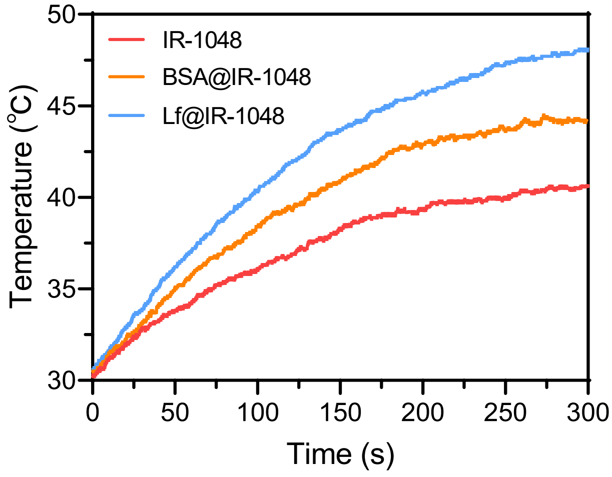

Figure S11. The photothermal conversion curves of IR-1048, BSA@IR-1048, and Lf@IR-1048 in PBS with concentration of 10 μM and 1064 nm laser (0.5 W/cm^2).

Calculation of the Photothermal Conversion Efficiency.

Following Roper's report (J. Phys. Chem. C 2007, 111, 3636), the total energy balance for the system can be expressed by Eq. 1:

$$\Sigma_i m_i C_{p,i} \frac{dT}{dt} = Q_{NC} + Q_{Dis} - Q_{surr} \quad (1)$$

Where m and C_p are the mass and heat capacity of water, respectively, T is the solution temperature, Q_{NC} is the energy inputted by NCs, Q_{Dis} is the baseline energy inputted by the sample cell, and Q_{surr} is heat conduction away from the system surface by air.

The laser-induced source term, represents heat dissipated by electron-phonon relaxation of the plasmons on the AMP surface under the irradiation of 1064 nm laser:

$$Q_{NC} = I(1 - A^{-1064})\eta \quad (2)$$

Where I is incident laser power, η is the conversion efficiency from incident laser energy to thermal energy, and A_{1064} is the absorbance of the probes at wavelength of 1064 nm. In addition, source term, Q_{Dis}, expresses heat dissipated from light absorbed by the quartz sample cell itself. Q_{surr} is linear with temperature for the outgoing thermal energy, as given by Eq. 3

$$Q_{surr} = hS(T - T_{surr}) \quad (3)$$

Where h is heat transfer coefficient, S is the surface area of the container, and T_{Surr} is ambient temperature of the surroundings.

Once the laser power is defined, the heat input (Q_{NC} + Q_{Dis}) will be finite. Since the heat output (Q_{Surr}) is increased along with the increase of the temperature according to the Eq. 3, the system temperature will rise to a maximum when the heat input is equal to heat output:

$$Q_{NC} + Q_{Dis} = Q_{surr-max} = hS(T_{max} - T_{surr}) \quad (4)$$

Where the $Q_{surr-max}$ is heat conduction away from the system surface by air when the sample cell reaches the equilibrium temperature, and T_{max} is the equilibrium temperature. The 1064 nm laser heat conversion efficiency (η) can be determined by substituting Eq. 2 for Q_{NC} into Eq. 4 and rearranging to get

$$\eta = \frac{hS(T_{max}-T_{surr})-Q_{Dis}}{I(1-10^{-A_{1064}})} \quad (5)$$

In order to get the hS, a dimensionless driving force temperature, θ is introduced using the maximum system temperature, T_{max}

$$\theta = \frac{T-T_{surr}}{T_{max}-T_{surr}}$$

(6)

and a sample system time constant τ_s,

$$\tau_s = \frac{\Sigma_i m_i C_{p,i}}{hS}$$

(7)

which is substituted into Eq. 1 and rearranged to yield

$$\frac{d\theta}{dt} = \frac{1}{\tau_s}\left[\frac{Q_{NC}+Q_{Dis}}{hS(T_{max}-T_{surr})} - \theta\right]$$

(8)

At the cooling stage of the aqueous dispersion of the sample, the light source was shut off, the Q_{NC} $+Q_{Dis} = 0$, reducing the Eq. 9

$$dt = -\tau_s \frac{d\theta}{\theta} \quad (9)$$

and integrating, giving the expression

$$\tau = -\tau_s \ln\theta \quad (10)$$

finally, find the value of photothermal conversion efficiency (η)

$$\eta = \frac{hS(T_{max}-T_{surr})-Q_{Dis}}{I(1-10^{-A_{1064}})}$$

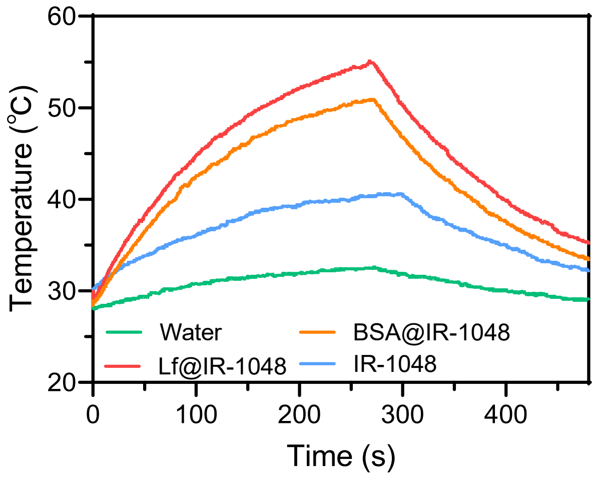

Figure S12. Photothermal effect of PBS, IR-1048, BSA@IR-1048, and Lf@IR-1048 (10 μM) under irradiation of 1064 nm (0.5 W/cm^2) for 270 s and then cooling down.

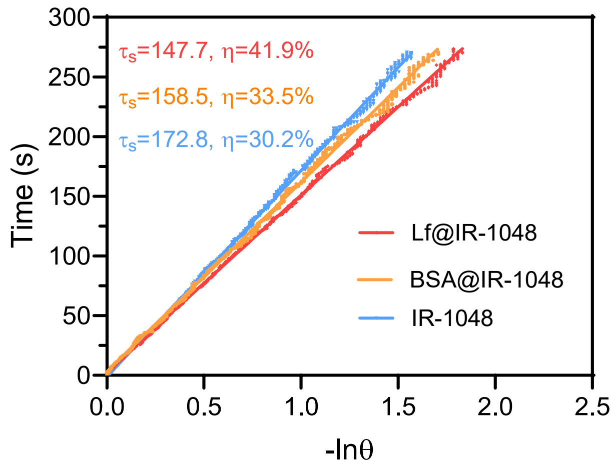

Figure S13. The relationship between the negative natural logarithm of the temperature change and the cooling time after laser irradiation.

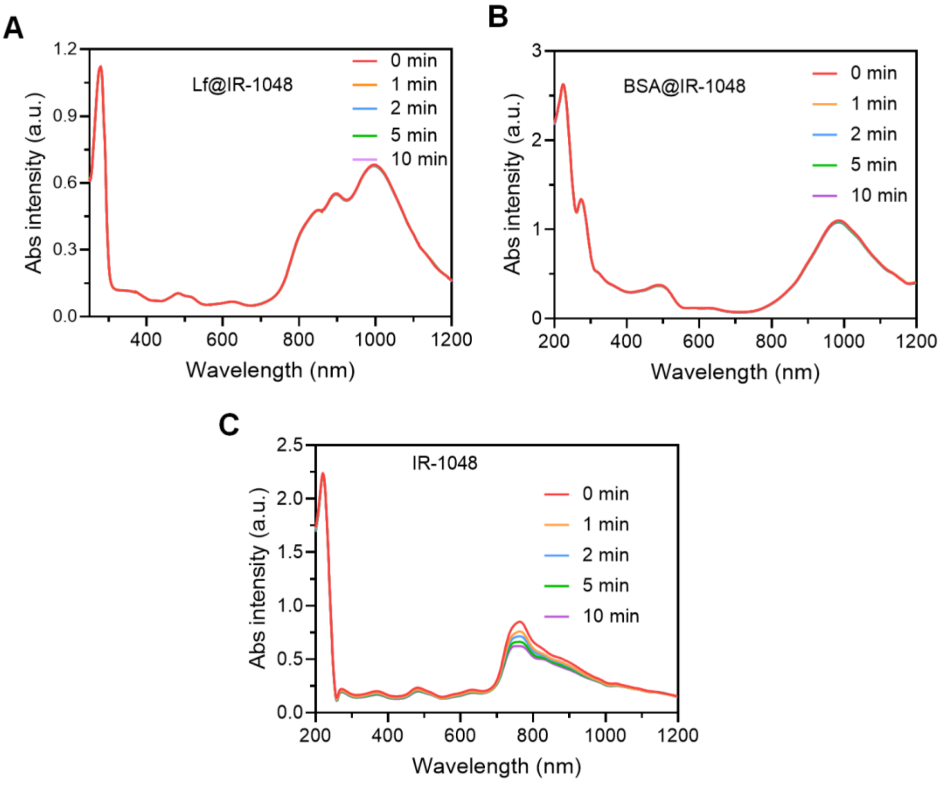

Figure S14. The UV-vis-NIR spectra of Lf@IR-1048 (A), BSA@IR-1048 (B), and IR-1048 (C) after continuous laser radiation.

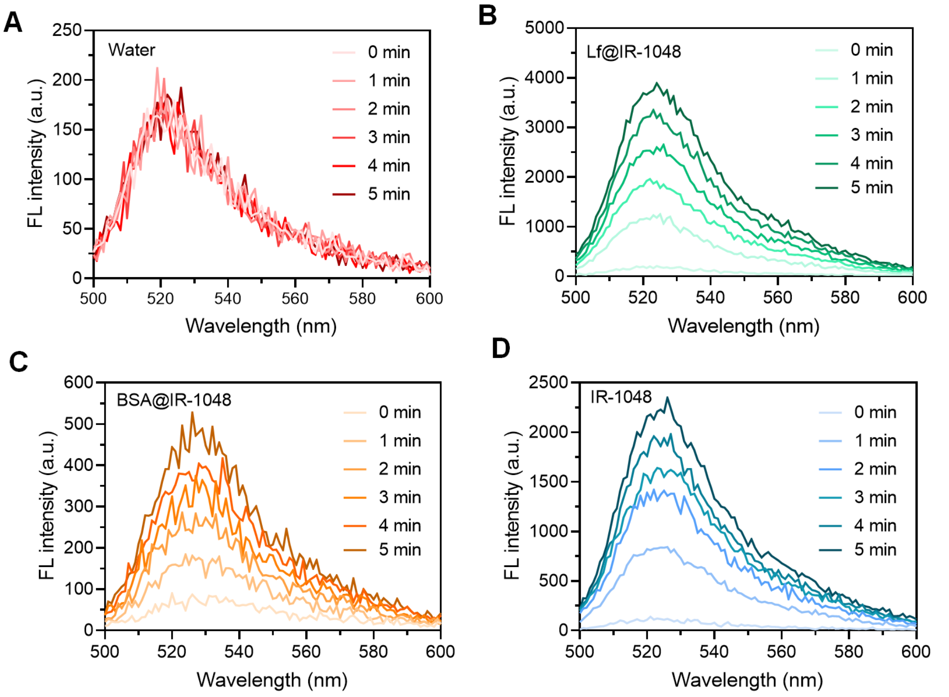

Figure S15. The fluorescence spectra of DCFH-DA treated PBS (A), IR-1048 (B), BSA@IR-1048 (C), and Lf@IR-1048 (D) under 1064 nm laser irradiation (0.5 W/cm^2) for 5 min.

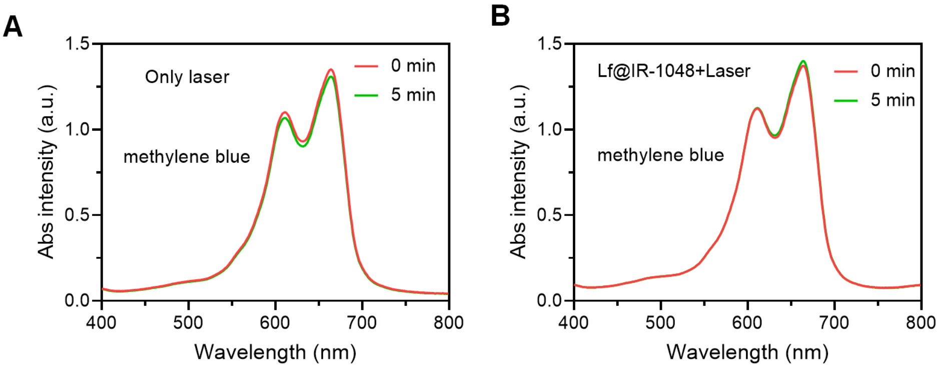

Figure S16. The absorption spectra of MB after treated with laser (A) and Lf@IR-1048 + Laser (B).

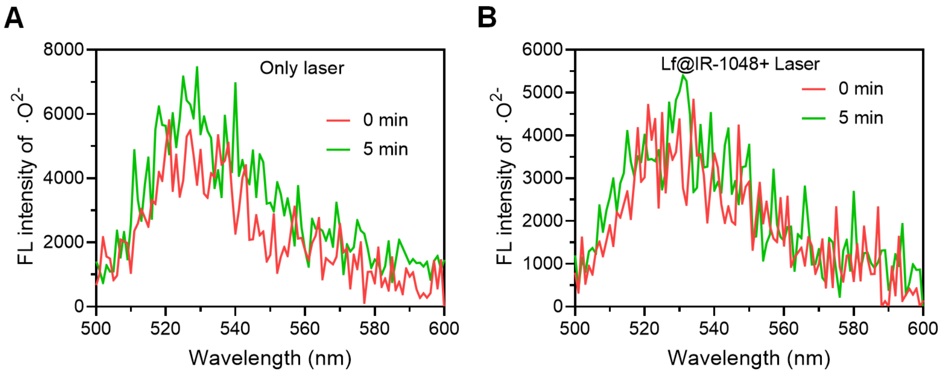

Figure S17. The fluorescence spectra of DHR123 after treated with laser (A) and Lf@IR-1048 + Laser (B).

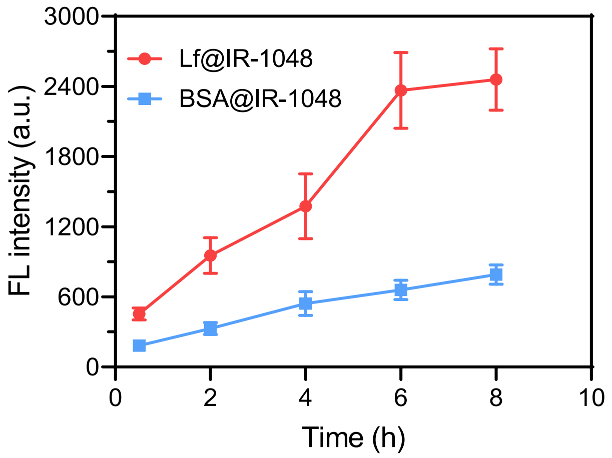

Figure S18. Quantification the FL intensity of Lf@IR-1048 and BSA@IR-1048 in CT26 cells at different time-points.

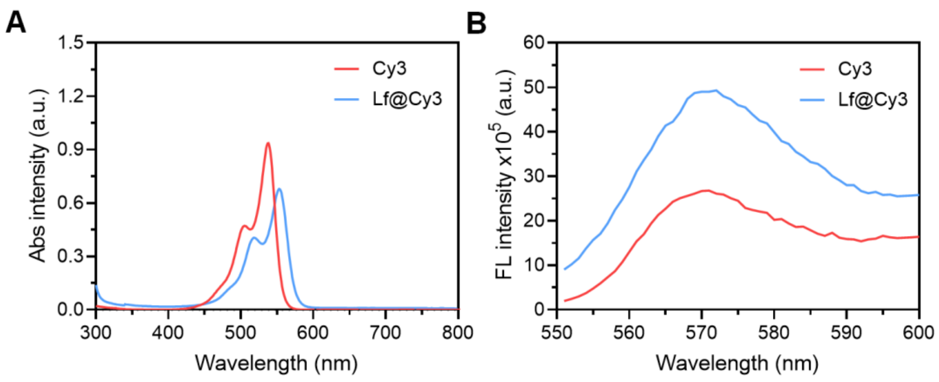

Figure S19. (A) UV-vis-NIR spectra of Cy3 and Lf@Cy3 in water. (B) Fluorescence spectra of Cy3 and Lf@Cy3 in water under excitation wavelength of 550 nm.

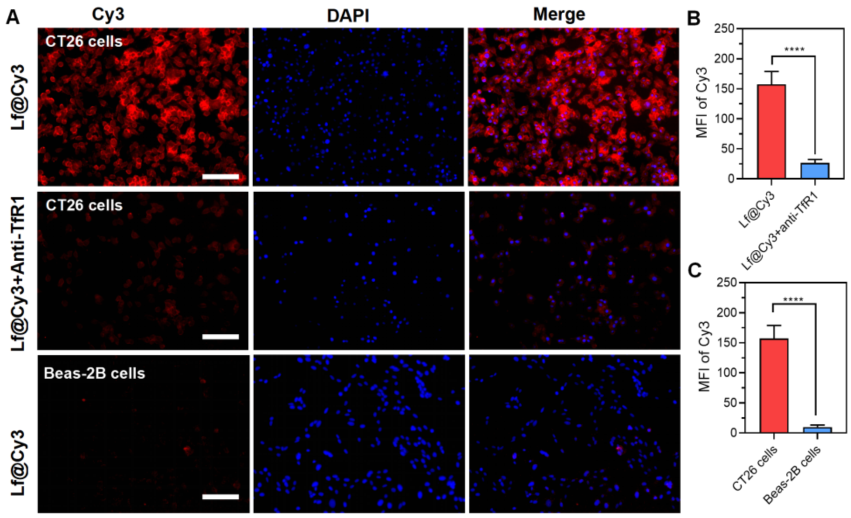

Figure S20. (A) Fluorescence images and quantitative analysis of CT26 and Beas-2B cells uptake of the Lf@Cy3 complex with or without Anti-TfR1. (B) Fluorescence quantification of CT26 cells uptake of the Lf@Cy3 complex with or without Anti-TfR1 (Scale bar: 100 μm). (C) Fluorescence quantification of CT26 cells and Beas-2B cells uptake of the Lf@Cy3 complex. ****$P < 0.0001$.

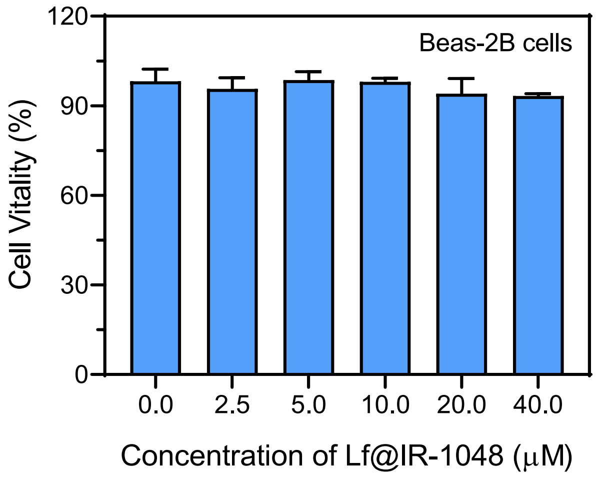

Figure S21. Cell viability of Beas-2B cells was assessed by CCK-8 assay after incubation with Lf@IR-1048 at various concentrations ranging from 0 to 40 μM.

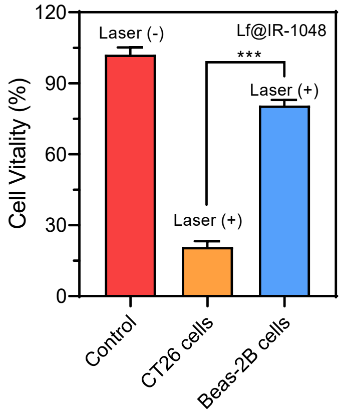

Figure S22. Cell viability of CT26 cells and Beas-2B cells treated with Lf@IR-1048 + Laser. ***P < 0.001.

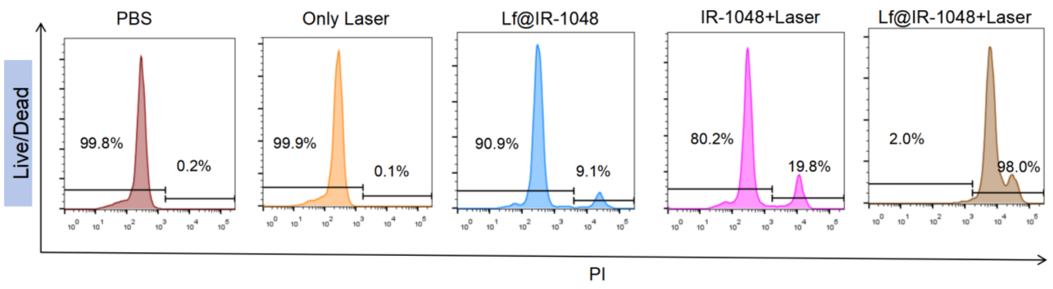

Figure S23. The results of live/dead cells by flow cytometry after different treatments. (All groups: PBS, Only laser, Lf@IR-1048, IR-1048 + Laser, Lf@IR-1048 + Laser).

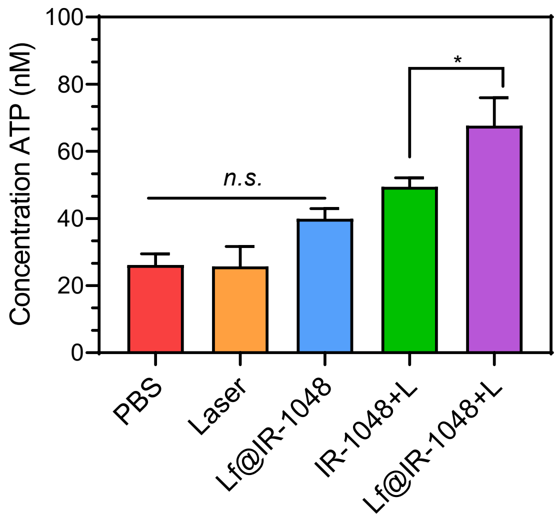

Figure S24. The ATP in the cellular supernatant measured by ELISA kits after treated with different groups. *P < 0.05. n.s., no significant difference between two groups.

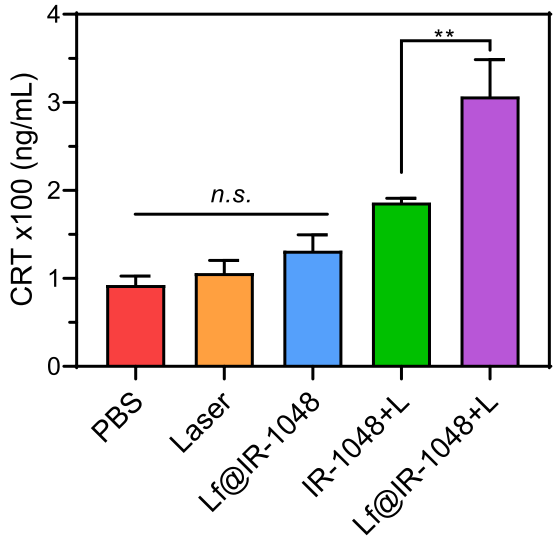

Figure S25. The CRT in the cellular supernatant measured by ELISA kits after treated with different groups. **P < 0.01. n.s., no significant difference between two groups.

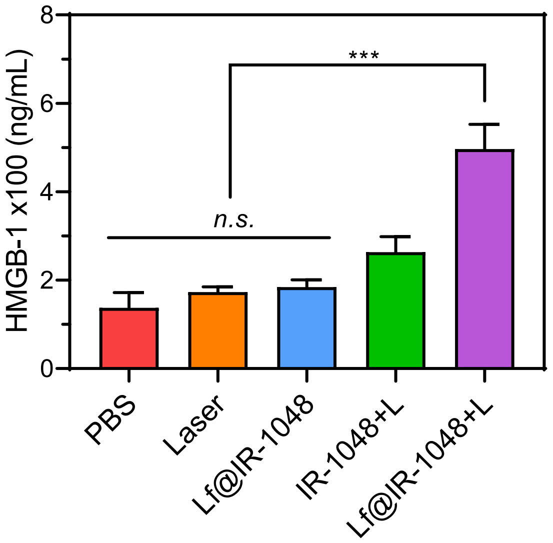

Figure S26. The HMGB1 in the cellular supernatant measured by ELISA kits after treated with different groups. ***P < 0.001. n.s., no significant difference between two groups.

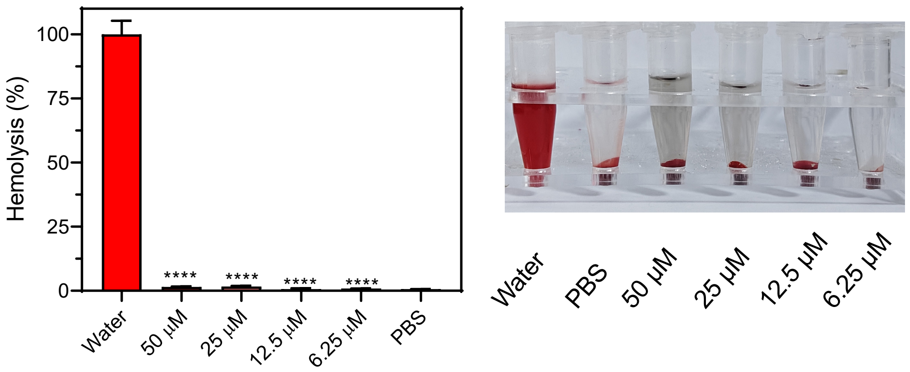

Figure S27. A hemolysis assay was applied to investigate the biosecurity of Lf@IR-1048.

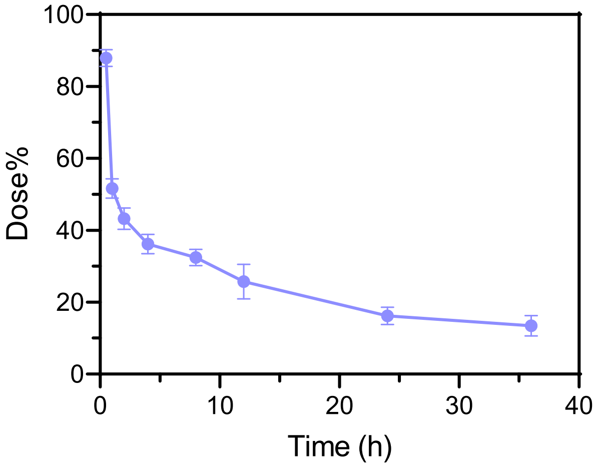

Figure S28. The pharmacokinetics of Lf@IR-1048 at different time points.

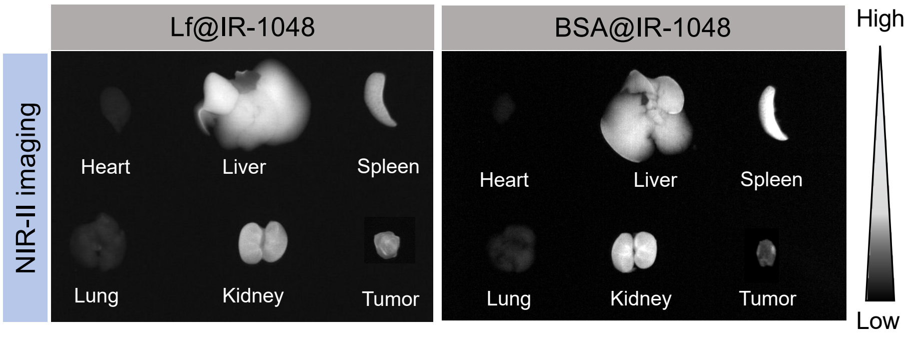

Figure S29. *Ex vivo* NIR-II fluorescence imaging of tumors and organs of mice collected from Lf@IR-1048 and BSA@IR-1048 groups under excitation wavelength of 980 nm.

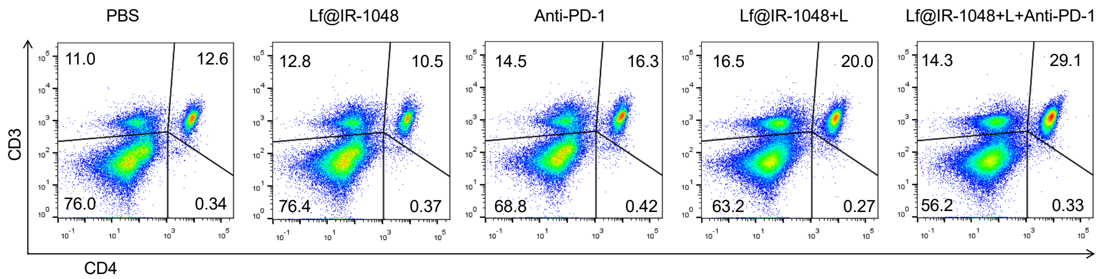

Figure S30. The percentage of $CD4^+$ T cells in leukocytes with CD3 expression stimulated with different treatments and measured by flow cytometry.

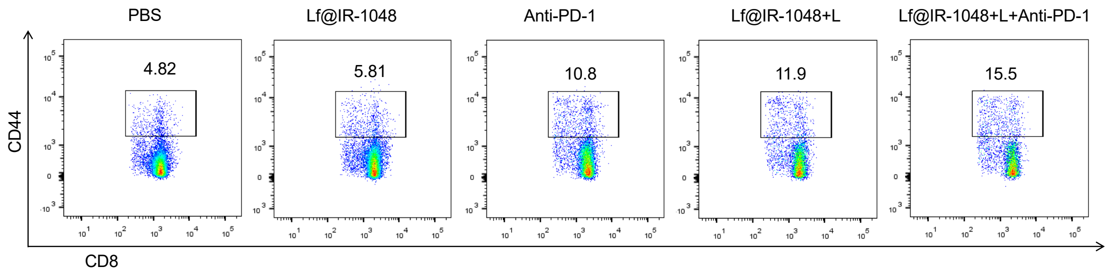

Figure S31. The percentage of memory $CD8^+$ T cells with the expression of CD44 after different treatments.
